# Plasma Proteomic Changes of Atherosclerosis after Exercise in ApoE Knockout Mice

**DOI:** 10.3390/biology11020253

**Published:** 2022-02-06

**Authors:** Chen-Chung Liao, Jin-Wei Xu, Wen-Ching Huang, Hung-Chang Chang, Yu-Tang Tung

**Affiliations:** 1 Metabolomics-Proteomics Research Center, National Yang Ming Chiao Tung University, Taipei 112, Taiwan; ccliao@nycu.edu.tw; 2 Department of Forestry, National Chung Hsing University, Taichung 402, Taiwan; ecsgunro@gmail.com; 3 Department of Exercise and Health Science, National Taipei University of Nursing and Health Sciences, Taipei 112, Taiwan; wenching@ntunhs.edu.tw; 4 Graduate Institute of Biotechnology, National Chung Hsing University, Taichung 402, Taiwan; benny19981204@gmail.com; 5 Nutrition Research Center, Taipei Medical University Hospital, Taipei 110, Taiwan; 6 Cell Physiology and Molecular Image Research Center, Wan Fang Hospital, Taipei Medical University, Taipei 116, Taiwan

**Keywords:** atherosclerosis, complement factor C5, exercise, proteomic changes, chemotaxis

## Abstract

**Simple Summary:**

A proteomic approach was applied to investigate the molecular mechanism of exercise on atherosclerosis. The MYOCD, PROS1, C2, SERPINA10, CRP, F5, C5, CFB, FGG, CFH, F12, PRDX2, PROZ, PPIA, and HABP2 levels associated with cardiovascular disease induced by a Western diet (WD) were significantly decreased by exercise intervention. Furthermore, the downregulation of complement factor C5 expression in the aortic root results in a decrease in macrophage infiltration of cholesterol-driven plaques. Therefore, exercise can help mitigate the atherosclerosis by ameliorating complement system activation and inflammatory responses in the aorta.

**Abstract:**

Atherosclerosis is the preliminary cause of coronary artery disease, one of the diseases that account for the largest number of fatal mortalities. Physical activity is an effective strategy to restrain atherosclerosis from deterioration. Evidence indicated that changes in the proteomic profile are highly associated with atherosclerosis development, but the mechanism behind exercise for atherosclerosis amelioration has not yet been investigated from a proteomics perspective. Hence, the proteomic profiles could further elucidate the systematic effects of exercise intervention on ApoE knockout atherosclerotic model and high-fat-diet intervention. In the current study, Apoe^em1Narl^/Narl mice were randomly allocated into a normal diet (ND), Western diet (WD), and WD with 12-week exercise intervention (WD EX) groups. The plasma proteome between WD and WD EX groups demonstrate the significant difference, and ten major pathways, including cardiovascular disease (CVD)–hematological disease, inflammatory disease, infectious diseases, inflammatory response, cell-to-cell signaling and interaction, connective tissue disorders_inflammatory disease, metabolic disease_organismal injury and abnormalities, cell-to-cell signaling and interaction, connective tissue disorders_inflammatory disease, and endocrine system disorders_gastrointestinal disease, etc., were generated by the IPA analysis. The 15 proteins (MYOCD, PROS1, C2, SERPINA10, CRP, F5, C5, CFB, FGG, CFH, F12, PRDX2, PROZ, PPIA, and HABP2) critically involved in CVD–hematological disease pathway showed significant difference between WD and WD EX groups. In current study, exercise could significantly alleviate the significantly elevated C5 and inflammation induced by the WD group in accordance with amelioration of atherosclerosis. Therefore, exercise could mitigate chemotaxis through the modulation of the C5 level and innate immunity, thereby alleviating the pathogenesis of atherosclerosis in Western-diet-induced obese mice.

## 1. Introduction

Cardiovascular disease (CVD) is the most life-threatening chronic disease according to the statistics of World Health Organization. CVD claimed an estimated 17.9 million deaths every year (approximately 31% of all global deaths), making it the most notorious life-threatening illness in the past 15 years. Atherosclerosis, a chronic and accelerating disease caused by the progression of atheromatous plaque accumulation in the vascular endothelium, is the main cause of CVD [1,2]. CVD is the most common cause of acute cardiovascular events such as myocardial infarction and stroke [3].

After the first diagnosis of CVD or recurrent vascular events, the strategy of a healthy lifestyle and exercise is the first and secondary prevention [4]. Behavioral modification could reduce up to 80% of coronary heart diseases [5,6], and an estimated 75% of recurrent vascular events may be prevented by medication when combined with lifestyle changes. Exercise and physical activities effectively reduce the risk of atherosclerosis [7]. Exercise regulates blood lipid homeostasis, which could ameliorate the pathogenesis of atherosclerosis. Moreover, exercise could also lower blood glucose levels and reduce the risk of obesity and diabetes for multiple risks modulation [8]. Exercise also demonstrated a positive effect on blood pressure management, a well-known risk factor of atherosclerosis, through promoting the nitric oxide secretion from endothelial cells and maintenance of vascular relaxation [9]. Hence, regular exercise may minimize multiple risks for atherosclerosis.

The whole pathogenesis of atherosclerosis is divided into three stages: the initiation of lesions, fatty streak formation, and advanced atheromatous plaque formation. Finally, the disease progresses into atherothrombosis [10]. Risk factors such as age, gender, blood pressure, blood cholesterol levels, obesity, and smoking are known to elevate the possibility of developing atherosclerosis [11,12]. These well-established risk factors are classified into three different categories, including lipoprotein, inflammation, and coagulation markers, and they have been identified as a form of risk prediction in the diagnosis of atherosclerosis [11]. However, there are no accurate markers to identify the exact lesion stages in the development of atherosclerosis according to current research.

Recently, apolipoprotein E knockout mice have been shown to develop severe hypercholesterolemia and atherosclerotic lesions that are more characteristic in appearance and distribution than those observed in humans [13]. Apolipoprotein E knockout mice on a high-fat diet have been shown to be closely associated with the development of atherosclerosis, which is characterized by excessive accumulation of TC and LDL-C in the vessel wall and is considered ideal for an animal model for atherosclerosis research [14]. Therefore, the present study aimed to evaluate the protective effects of exercise on the development of atherosclerosis and confirm the potential effective proteins with exercise intervention during the atherosclerosis through proteomic changes in the plasma in ApoE knockout atherosclerotic model and high-fat-diet intervention.

## 2. Materials and Methods

### 2.1. Animal Model

The study was conducted under strict guidelines of the Institutional Animal Care and Use Committee of Taipei Medical University (approval number: LAC-2017-0230). A total of 18 male apolipoprotein E knockout mice (Apoe^em1Narl^/Narl, ApoE knockout) with C56BL6 backgrounds were purchased form the National Laboratory Animal Center (Taipei, Taiwan) at 6 weeks of age. Animals were housed in the barrier-controlled Laboratory Animal Center (temperature: 22 ± 2 °C, humidity: 60%, 12 h light/dark cycle). All mice had free access to water and a basic diet (#5001, Laboratory rodent diet, LabDiet, St. Louis, MO, USA) for one week. After a week of acclimatization, the mice were randomly divided into three groups (*n* = 6). A normal diet group (ND group) was fed a normal diet (ND, #5001, Laboratory rodent diet, LabDiet, St. Louis, MO, USA) which is composed of 23% protein, 4.5% fat, and 6% fiber. The other two groups were fed with a Western diet (WD, #D12079B, Research Diets, New Brunswick, NJ, USA) which is composed of 41% fat (butterfat and corn oil) and 29% sucrose, combined with and without simultaneous exercise intervention, namely WD and WD EX groups, respectively. All animals were fed ad libitum for 12 weeks. Mice in the WD EX group were subjected to a 12-week swimming intervention according to a previous study [15] with slight modifications. Briefly, this involved 40 min of continuous swimming training in a temperature-controlled (35–36 °C) water bath 5 days a week. After 12 weeks of the experiment, all mice were euthanized with 2–2.5% isoflurane and the blood was collected through retro-orbital bleeding for proteomic analysis. The hearts were collected and fixed in 4% formaldehyde for morphological analysis of atherosclerotic plaque.

### 2.2. Morphological Analysis of Atherosclerotic Plaque

The aortic root dissection of mice was fixed with 10% formaldehyde and paraffin embedded. Tissue sections were observed and photographed under Masson’s trichrome and hematoxylin and eosin. Stained slides were digitalized using a Hamamatsu NanoZoomer (C9600-12) using NDP Scan software (all from Hamamatsu Photonics, Hamamatsu City, Japan). Total macrophage counts within plaques were measured by a clinical pathologist.

### 2.3. Proteomic Analysis

Plasma was digested with a SMART Digestion Trypsin Kit (P/N60109-101, Thermo Fisher, Bedford, MA, USA). The supernatant was denatured with dithiothreitol (DTT) at 58 °C for 30 min. Protein lysate was alkylated in the dark with iodoacetamide (IAA) at room temperature for 30 min. The peptide pool was then subjected to desalting and purification using ZipTip C18 SPE pipette tips (Millipore ZipTips Micro-C18, P/NZ720003, Sigma, St. Louis, MO, USA) and SOLA SPE Plates (Thermo Fisher, Bedford, MA, USA), respectively. Prior to analysis, the extracted tryptic peptides were diluted with 0.1% formic acid for subsequent analysis using an LTQ-Orbitrap Elite mass spectrometer (Thermo Electron, Waltham, MA, USA). The mass spectrometer was inline and coupled with a nanoACQUITY UPLC system (Waters, Milford, MA, USA), equipped with a C18 trap column (5 μm, 180 μm × 20 mm, Symmetry C18, Waters, Milford, MA, USA) and a BEH130 C18 separation column (1.7 μm, 100 μm × 100 mm, Waters, Milford, MA, USA). The gradient elution of peptides in nanoLC was from 3 to 40% acetonitrile (ACN) for 168 min, 40 to 95% ACN for 2 min, and 95% ACN for 10 min, all in 0.1% aqueous solution of formic acid at a flow rate of 0.3 mL/min. The eluted peptides were ionized with a spray voltage of 2.33 kV and introduced into the LTQ-Orbitrap Elite mass spectrometer. Mass spectrometry was conducted in the positive ion mode and based on a data-dependent acquisition method (isolation width: 1.5 Da). Peptide mass spectrometry data were obtained using a full mass spectrometer survey scan (m/z range of 350–1600) with 30,000 resolution at *m*/*z* 400. The top 15 most intensively charged peptide ions were scanned according to the data-dependent acquisition method. The selected precursor peptide ions were stimulated with helium collision-induced dissociation (CID) of the selected precursor peptide ions. The UniProt mouse protein database (containing 17,089 protein sequences; released on July 2021; http://www.uniprot.org/) was used to define the acquired proteomics raw data files with PEAKS Studio 7.5 (Bioinformatics Solutions, Waterloo, ON, Canada). A protein was identified when matched with at least one unique peptide—quantitative analysis of proteins by MS spectra was counted with in-house software [16,17]. With a 1% false discovery rate (FDR), a total of 961 and 939 proteins were identified in the first group (ND vs. WD) and second group (WD vs. WD EX), respectively. Spectrum counts were normalized with the total identified spectra per biological sample and the proteins. A t test was performed to determine the significance of proteins. State-of-the-art Ingenuity Pathway Analysis (IPA, Ingenuity Systems Inc., Redwood City, CA, USA) software application was used to reveal the global network functions of all proteins differentially expressed. Accession numbers and expression fold changes of the proteins were uploaded into the IPA software application for interaction network and biological function grouping for different protein expressions. Fisher’s exact test was used to calculate the values at which *p* was significant. Moreover, the mass spectrometry proteomics data have been deposited to the ProteomeXchange Consortium via the PRIDE [18] partner repository with the dataset identifier PXD030951 and 10.6019/PXD030951.

### 2.4. Immunohistochemical Examination

Formaldehyde-fixed and paraffin-embedded sections of the hearts were dissected to a thickness of 4 μm. The sections were incubated in ethylenediamineteraacetate buffer (pH 9, epitope retrieval solution) for antigen retrieval and in 0.02% H_2_O_2_ to block endogenous peroxidase activity. Then, the sections were incubated overnight at 4 °C with primary rabbit monoclonal antibody against complement C5 (PA2308, Boster, CA, USA, 1:1000) and then horseradish peroxidase–conjugated secondary antibody was used. Diaminobenzidine (DAB) was used for staining development and the sections were counter-stained with hematoxylin. The negative control consisted of substituting normal serum for primary antibody. The positive staining areas were quantified using ImageJ Fiji software version 12, and the integrated optical density (IOD) per stained area (IOD/area) was calculated.

### 2.5. Statistical Analysis

The results were presented as means ± standard deviation (*n* = 6). Significant differences were calculated using *t* test, Fisher’s exact test or Tukey’s post hoc test with GraphPad Prism version 6.0 or SAS version 9.4. All results with *p* < 0.05 were considered to be statistically significant.

## 3. Results

### 3.1. The Effect of Exercise on the Development of Atherosclerotic Plaque

**Figure 1:**
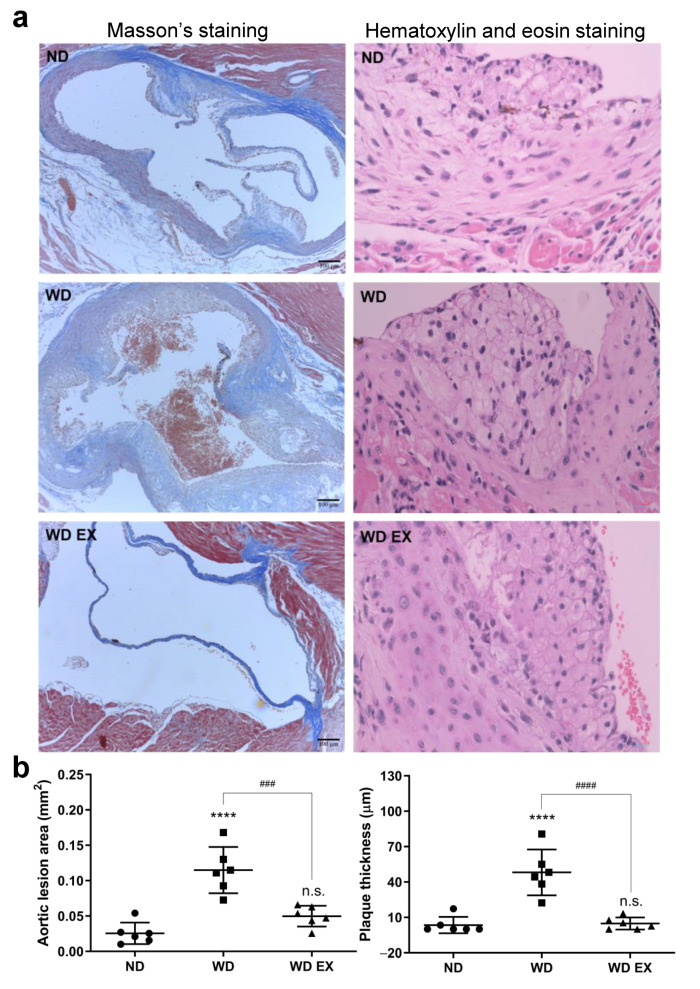
Effects of exercise on (**a**) Masson’s staining and hematoxylin and eosin staining, and (**b**) quantification of the aortic lesion area and plaque thickness in ApoE knockout mice. The results are expressed as means ± standard deviation (*n* = 6). One-way ANOVA followed by Tukey’s post hoc test was used for statistical analysis, and **** *p* < 0.0001 represents the significance between ND and WD groups. ^###^ *p* < 0.001 and ^####^ *p* < 0.0001 represents the significance between WD and WD EX groups. n.s. = No significant difference.

After 12 weeks of WD diet and exercise intervention, the exercise significantly ameliorated the WD-induced obesity, but food intake and calorie intake were not affected (Supplementary Figure S1). To confirm atherosclerosis development, the Masson’s trichrome and hematoxylin and eosin staining of aortic sinus sections in the ND, WD, and WD EX groups were shown in Figure 1a. In the WD group, a greater amount of atherosclerotic plaque areas and cholesterol plaque thicknesses was observed at the aortic root than the ND group (*p* < 0.05) and this indicated that cholesterol-driven advanced fibrous plaques formed after the WD intervention. Compared with the WD group, the area of atherosclerotic plaque and the thicknesses of cholesterol plaque in WD EX group were significantly ameliorated (*p* < 0.05). Therefore, exercise can prevent the formation of collagen-rich plaque, reduce aorta lesion area and plaque thickness, and further decrease the thickness of cholesterol plaques at the aortic root compared with the WD group.

### 3.2. The Effect of Exercise on Plasma Proteomic Changes

The plasma proteomic characteristics were analyzed by liquid chromatography-tandem mass spectrometry (LC-MS/MS) to discover proteins that may be associated with atherosclerosis. Table 1 showed 62 proteins were significantly different between WD and WD EX groups. Among them, 54 proteins in the WD group were significantly higher than those in the WD EX group, and 4 proteins were unique proteins to WD mice.

### 3.3. Effects of Exercise on Plasma Protein–Regulated Biofunction Pathways

**Figure 2:**
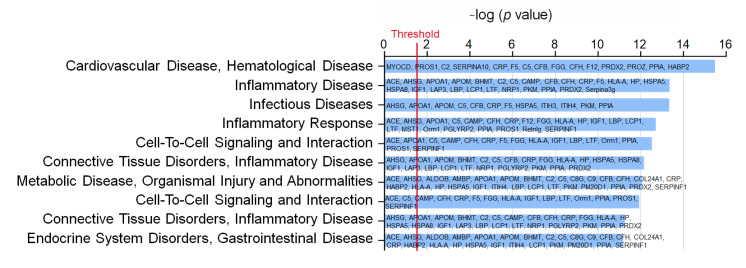
Effects of exercise on the biofunction pathway regulated by proteins between WD and WD EX groups. The 10 most different biofunction pathways are presented and ranked by significant differences between groups. The vertical line indicates the threshold at *p* < 0.05.

**Figure 3:**
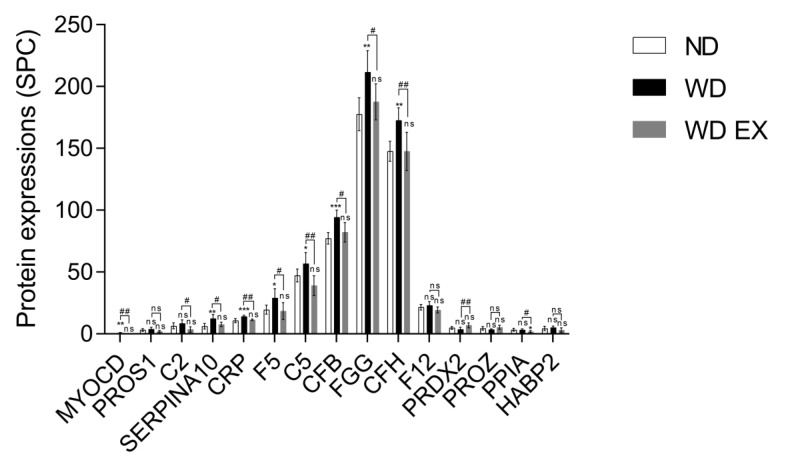
Effects of exercise on cardiovascular and hematological-disease-regulated proteins in ApoE knockout mice. The results are expressed as means ± standard deviation (*n* = 6). One-way ANOVA followed by Tukey’s post hoc test was used for statistical analysis, and * *p* < 0.05, ** *p* < 0.01, and *** *p* < 0.001 represents the significance between ND and WD groups or ND and WD EX groups. ^#^ *p* < 0.05 and ^##^ *p* < 0.01 represents the significance between WD and WD EX groups. ns = No significant difference.

To understand the impact of WD and WD EX groups on the changes of plasma proteomic, IPA was performed to clarify the proteins implicated in biofunction pathways (Supplementary Table S1). By applying the *p*-value of < 0.05 threshold, the pathway analysis from IPA provided 10 pathways (e.g., CVD_hematological disease, inflammatory disease, infectious diseases, inflammatory response, cell-to-cell signaling and interaction, connective tissue disorders inflammatory disease, metabolic disease_organismal injury and abnormalities, cell-to-cell signaling and interaction, connective tissue disorders_inflammatory disease, and endocrine system disorders_gastrointestinal disease) (Figure 2). Among the ten pathways, the detected proteins are mainly related to CVD and inflammation. The pathway with the smallest *p* value among these pathways is the CVD–hematological disease pathway, which involved 15 proteins, namely MYOCD, PROS1, C2, SERPINA10, CRP, F5, C5, CFB, FGG, CFH, F12, PRDX2, PROZ, PPIA, and HABP2 (Figure 2). Compared with the ND group, MYOCD, SERPINA10, CRP, F5, C5, CFB, FGG, and CFH levels were found to be significantly increased in the WD group (Figure 3). However, exercise significantly decreased these eleven proteins compared with the WD group. Moreover, MYOCD, SERPINA10, CRP, F5, C5, CFB, FGG, and CFH proteins also demonstrated a significant reduction in the WD EX group compared with the WD group. The role of complement factor C5 in the development of atherosclerosis during exercise is unclear. Therefore, in the study the effect of exercise on complement factor C5 in the development of atherosclerosis was deeply investigated.

### 3.4. Effects of Exercise on Complement Factor C5 in the Aortic Root

**Figure 4:**
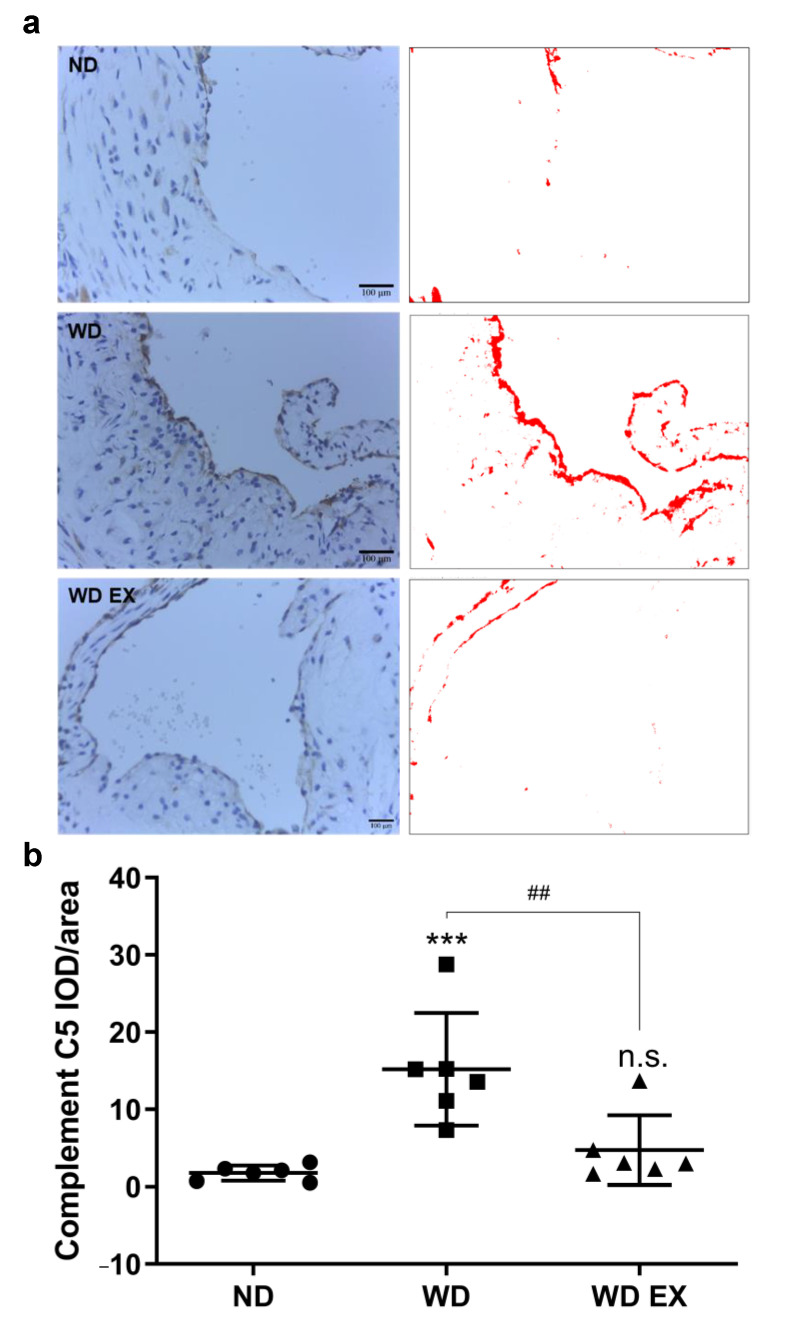
Effects of exercise on (**a**) aortic complement factor C5 expression and (**b**) quantitative analysis of the complement C5 IOD/area in ApoE knockout mice. Quantification of complement C5 staining and representative images. DAB-specific threshold selection (in red) from selected aortic root areas was performed using ImageJ, and total selective area was quantified and statistically analyzed. One-way ANOVA followed by Tukey’s post hoc test was used for statistical analysis, and *** *p* < 0.001 represents the significance between ND and WD groups. ^##^ *p* < 0.01 represents the significance between WD and WD EX groups. n.s. = No significant difference.

There is increasing evidence that the complement system is activated by atherosclerotic plaques during the pathogenesis of atherosclerosis [19]. The immunochemical staining of complement factor C5 protein in the aortic root was presented in Figure 4. Compared with the ND group, the complement factor C5 protein in the atherosclerotic plaque formed at the aortic root of the WD group was significantly increased. Therefore, the complement factor C5 may play an important role in the pathogenesis of atherosclerosis. However, WD EX group reduced the increase in complement factor C5 compared with WD group.

### 3.5. Effects of Exercise on Macrophage Infiltration of the Cholesterol-Driven Plaque Formed in the Aortic Root

**Figure 5:**
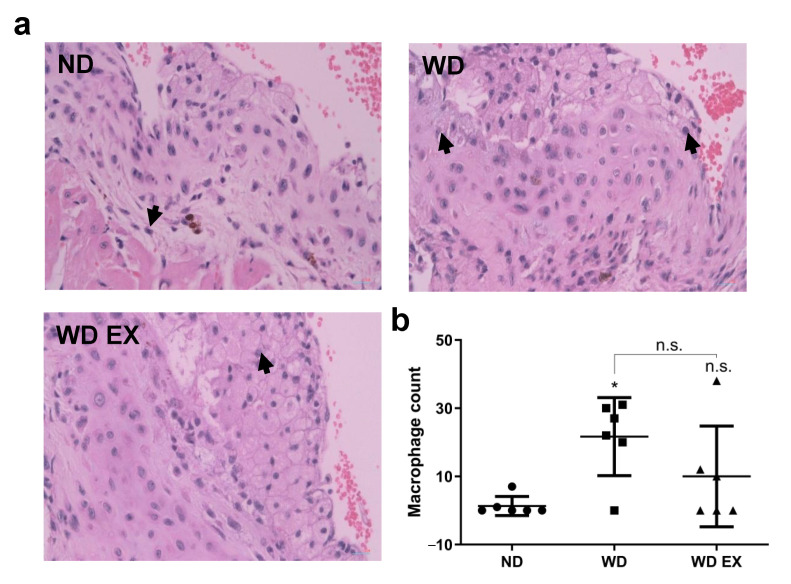
Effects of exercise on (**a**) macrophage infiltration in cholesterol plaque, the black arrows mean the macrophage infiltrated in the plaque and (**b**) quantitative analysis of macrophage count in plaque in ApoE knockout mice. The results are expressed as means ± standard deviation (*n* = 6). One-way ANOVA followed by Tukey’s post hoc test was used for statistical analysis, and * *p* < 0.05 represents the significance between ND and WD groups. n.s. = No significant difference.

The histological staining of macrophages within the cholesterol plaque formed in the endothelium of the aortic root was shown in Figure 5. The ND group exhibited the lowest macrophage counts, indicating the mild atherosclerotic plaque was formed in the aortic root of ND group. However, WD group increased inflammation in the plaque site and the macrophage counts of cholesterol-dense fibrous plaque in the aortic root. However, exercise slightly reduced the macrophage counts in atherosclerotic plaque, thereby alleviating plaque formation. These results indicated exercise can combat WD-induced atherosclerosis through inhibiting complement factor C5, alleviating macrophage accumulation in the inflammatory site and further suppressing the progression of atherosclerosis.

## 4. Discussion

Exercise has a deterrent effect on cardiovascular disease, and its anti-atherosclerotic effect has been described in different animal models [20,21]. Exercise can also positively affect risk factors associated with cardiovascular disease, hypertension, diabetes, obesity, increased plasma lipids, and endothelial dysfunction [22]. Exercise has anti-inflammatory effects to prevent the progression of atherosclerosis [15,23,24,25,26]. However, few studies have investigated how exercise affects circulatory protein levels in disease prevention or amelioration. To the best of our knowledge, the present study is the first to evaluate the protective effects of exercise on the development of atherosclerosis through proteomic changes in the plasma. Proteomics is a comprehensive study to understand the whole proteome or the sum of all proteins under the influence of different disease threats [27]. In the present study, the plasma proteomic changes of atherosclerosis in ApoE knockout mice after exercise were analyzed by LC-MS/MS. The proteomics changes between the WD and WD EX groups were analyzed, and the first 10 biological function pathways, namely CVD_hematological disease, inflammatory disease, infectious diseases, inflammatory response, cell-to-cell signaling and interaction, connective tissue disorders_inflammatory disease, metabolic disease_organismal injury and abnormalities, cell-to-cell signaling and interaction, connective tissue disorders_inflammatory disease, and endocrine system disorders_gastrointestinal disease, were involved with 62 differential protein expressions. As expected, most differential proteins are associated with CVD, inflammatory diseases and metabolic diseases, and these proteins were involved by complement factor proteins, coagulation factor proteins, and others. In the present study, the CVD–hematological disease biofunction pathway was further discussed.

The final stage of atherosclerosis is atherothrombosis [12]. Elevated coagulants such as fibrinogen (coagulation factor) and fibrin could increase the risk of atherothrombosis [28]. In the study, F5, FGG, and F12 were found to be significantly increased in the WD group that involved in CVD–hematological disease biofunction pathway belonging to the coagulation factor. These results indicated that WD may stimulate the occurrence of atherothrombosis in ApoE knockout mice. However, exercise significantly decreased F5 and FGG for potential amelioration of atherothrombosis in the pathogenesis of atherosclerosis, thereby suppressing disease progression.

The complement system is the main component of the innate immune system and a major factor in many chronic inflammatory diseases, such as atherosclerosis [19]. The complement system also promotes atherosclerosis in vessel walls by activating innate immunity [19]. In our study, the complement proteins (CRP, C5, CFB, and CFH) were found to be significantly increased in the WD group. These results indicated that WD-induced activation of innate immunity may accelerate the progression of atherosclerosis and lead to the formation of advanced fibrous plaque, which is consistent with previous studies [19,29,30,31]. C5a was found in cholesterol cracks and necrotic cell debris in cholesterol-driven inflammatory lesions, but C5a was almost undetectable in stable plaques retrieved by atherectomy in human coronary lesion areas [19]. Moreover, CFB simultaneously activated C3 and C5 to produce anaphylatoxins C3a and C5a, which further triggered the assembly of the terminal complement complex of inflammation [29]. CFB deficiency also demonstrated plaque reduction in mice fed high-fat diets [30]. Previous studies showed that CRP, a powerful risk biomarker of CVD, was detected in the intima of atherosclerotic plaques and colocalized with the terminal complement complex at the inflammatory site [31,32]. The amount of CFH was found to be much greater in the superficial layers than in the deep layers of human coronary atherosclerotic lesions, indicating a potential role for CFH in atherogenesis [33].

Exercise could also be considered as a stressor that can promote the acute breakdown of the stable immunity, leading to chronic adaptation [34]. Neutrophil dysfunction and impairment of the complement system appeared after exercise [35]. Our results showed that CRP, C5, CFB, and CFH in the blood were found to be significantly decreased in the WD EX group. Wolach et al. [36] showed that gymnasts have lower C2 and C3 compared with sedentary counterparts. However, the effects of exercise on complement factor C5 in the development of atherosclerosis have not been investigated. Therefore, in the study we found that WD can increase complement factor C5; however, exercise can reduce the increase in C5.

C5 is a key convertase for producing anaphylatoxins C5a and C5b. Thrombin can also induce the production of C5b, further aggravating the immune response [19,37,38]. In the present study, WD significantly increased plasma C5 and increased C5 in the fibrous plaque. These results are consistent with the results of previous studies, confirming the observation of C5 in cholesterol-rich inflammatory plaque lesions [19]. However, exercise significantly decreased the WD-induced elevation in C5 of plasma and plaque. The mild expression of C5 in the relatively stable plaque formed with lower collagen and elastin concentrations in the lesion, indicating that the activation of innate immunity was alleviated by exercise. Taken together, the results indicated that blood C5 levels may be a useful biomarker to reveal the onset and intervention efficacy for pathogenesis of atherosclerosis, and exercise could slow down the immune response for atherosclerosis.

**Figure 6:**
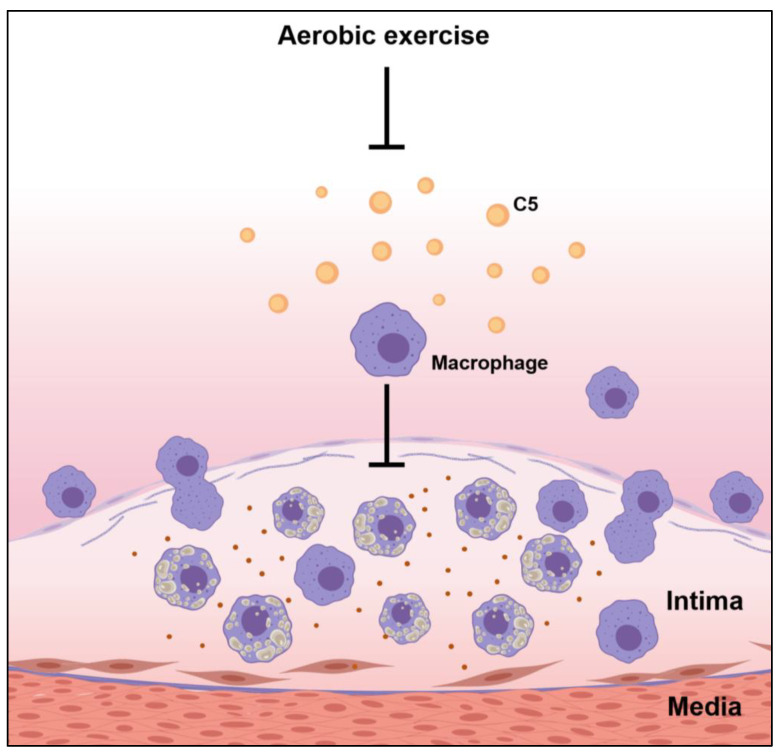
Possible effects of exercise on the pathogenesis of atherosclerosis.

Atherosclerosis is a chronic inflammation, and macrophages play an important role in atherosclerosis. Macrophages participate in the formation of unstable cholesterol-driven plaques in the highly pro-inflammatory microenvironment of the aortic endothelium, and the aortic endothelium fills the growing atherosclerotic lesions. Anaphylatoxins, particularly C3a and C5a, influenced macrophage responses by triggering the activation of inflammasomes and inflammation. Moreover, complement component C5 may interact with a variety of macrophage receptors, leading to the modulation of cytokine production as well as inflammatory responses [19]. Our results demonstrated that the ND group, with mild atherosclerotic symptoms, had the lowest macrophage counts in the plaque sites and the lowest C5 levels in the aortic root. In contrast, the WD group, with advanced fibrous plaque formation in the aortic root, had the highest C5 and macrophage infiltration in plaque, indicating that a WD triggered the activation of the complement system, increased complement factor C5 expression, and boosted macrophage counts in the endothelium site in the aortic root, thereby promoting the development of atherosclerosis. Exercise reduces the expression of C5 in the aorta, thereby downregulating the infiltration of macrophages, further improving the progression of atherosclerosis, and thus has a protective effect on the pathogenesis of atherosclerosis (Figure 6). In summary, these data further confirmed exercise can help fight atherosclerosis through downregulating the complement system.

## 5. Conclusions

Proteomics is the large-scale comprehensive study of proteins, including protein abundance and their interaction networks. In addition, proteomic analysis is a powerful tool to study protein expression changes and identify biomarkers of pathogenic processes. In this study, a proteomic approach was applied to investigate the molecular mechanism of exercise on atherosclerosis. The downregulation of complement factor C5 expression in the aortic root results in a decrease in macrophage infiltration of cholesterol-driven plaques. Therefore, exercise can help mitigate atherosclerosis by ameliorating complement system activation and inflammatory responses in the aorta.

## Figures and Tables

**Table 1:** The differentially proteins between WD and WD EX groups.

Accession No.	Gene ID	Protein Name	Respective Fold Change	*p*-Value
COOA1_MOUSE	Q30D77	Collagen alpha-1(XXIV) chain	-	-
HVM45_MOUSE	P01821	Ig heavy chain V region MC101	-	-
MYCD_MOUS	Q8VIM5	Myocardin	-	-
CP250_MOUSE	Q60952	Centrosome-associated protein CEP250	-	-
LOXL1_MOUSE	P97873	Lysyl oxidase homolog 1	−7.289	0.0220
DYHC1_MOUSE	Q9JHU4	Cytoplasmic dynein 1 heavy chain 1	−6.976	0.0232
AMPL_MOUSE	Q9CPY7	Cytosol aminopeptidase	−5.695	0.0125
NRP1_MOUSE	P97333	Neuropilin-1	−5.124	0.0163
BIP_MOUSE	P20029	Endoplasmic reticulum chaperone BiP	−4.781	0.0008
BHMT1_MOUSE	O35490	Betaine–homocysteine S-methyltransferase 1	−4.416	0.0157
PLSL_MOUSE	Q61233	Plastin-2	−3.783	0.0186
ACE_MOUSE	P09470	Angiotensin-converting enzyme	−3.692	0.0209
ALDOB_MOUSE	Q91Y97	Fructose-bisphosphate aldolase B	−3.373	0.0220
OBP1A_MOUSE	Q9D3H2	Odorant-binding protein 1a	−3.369	0.0127
SAP_MOUSE	Q61207	Prosaposin	−3.103	0.0206
RETNG_MOUSE	Q8K426	Resistin-like gamma	−3.020	0.0096
HSP7C_MOUSE	P63017	Heat shock cognate 71 kDa protein	−3.000	0.0117
HPT_MOUSE	Q61646	Haptoglobin	−2.745	0.0012
IGF1_MOUSE	P05017	Insulin-like growth factor I	−2.576	0.0395
PSA7_MOUSE	Q9Z2U0	Proteasome subunit alpha type-7	−2.451	0.0326
CO2_MOUSE	P21180	Complement C2	−2.355	0.0091
COL11_MOUSE	Q3SXB8	Collectin-11	−2.349	0.0378
KPYM_MOUSE	P52480	Pyruvate kinase PKM	−2.280	0.0093
LBP_MOUSE	Q61805	Lipopolysaccharide-binding protein	−2.251	0.0029
PROS_MOUSE	Q08761	Vitamin K-dependent protein S	−1.901	0.0336
PI16_MOUSE	Q9ET66	Peptidase inhibitor 16	−1.892	0.0270
PPIA_MOUSE	P17742	Peptidyl-prolyl cis-trans isomerase A	−1.787	0.0095
TRFL_MOUSE	P08071	Lactotransferrin	−1.771	0.0189
HGFL_MOUSE	P26928	Hepatocyte growth factor-like protein	−1.717	0.0059
HABP2_MOUSE	Q8K0D2	Hyaluronan-binding protein 2	−1.684	0.0435
A1AG2_MOUSE	P0736	Alpha-1-acid glycoprotein 2	−1.629	0.0198
ZPI_MOUSE	Q8R121	Protein Z-dependent protease inhibitor	−1.600	0.0222
FA5_MOUSE	O88783	Coagulation factor V	−1.582	0.0282
A1AG1_MOUSE	Q60590	Alpha-1-acid glycoprotein 1	−1.542	0.0097
ACTBL_MOUSE	Q8BFZ3	Beta-actin-like protein 2	−1.542	0.0029
CO5_MOUSE	P06684	Complement C5	−1.454	0.0050
HEMO_MOUSE	Q91 × 72	Hemopexin	−1.448	0.0074
CO8G_MOUSE	Q8VCG4	Complement component C8 gamma chain	−1.391	0.0092
PEDF_MOUSE	P97298	Pigment epithelium-derived factor	−1.369	0.0078
SAA4_MOUSE	P31532	Serum amyloid A-4 protein	−1.320	0.0164
ITIH4_MOUSE	A6 × 935	Inter alpha-trypsin inhibitor, heavy chain 4	−1.311	0.0134
PGRP2_MOUSE	Q8VCS0	N-acetylmuramoyl-L-alanine amidase	−1.304	0.0173
AMBP_MOUSE	Q07456	Protein AMBP	−1.304	0.0063
ITIH3_MOUSE	Q61704	Inter-alpha-trypsin inhibitor heavy chain H3	−1.277	0.0389
CRP_MOUSE	P14847	C-reactive protein	−1.246	0.0003
APOA1_MOUSE	Q00623	Apolipoprotein A-I	−1.236	0.0093
FA12_MOUSE	Q80YC5	Coagulation factor XII	−1.194	0.0425
ITIH2_MOUSE	Q61703	Inter-alpha-trypsin inhibitor heavy chain H2	−1.193	0.0103
CFAH_MOUSE	P06909	Complement factor H	−1.170	0.0094
CO9_MOUSE	P06683	Complement component C9	−1.158	0.0229
CFAB_MOUSE	P04186	Complement factor B	−1.147	0.0139
ITIH1_MOUSE	Q61702	Inter-alpha-trypsin inhibitor heavy chain H1	−1.144	0.0071
FETUA_MOUSE	P29699	Alpha-2-HS-glycoprotein	−1.141	0.0428
FIBG_MOUSE	Q8VCM7	Fibrinogen gamma chain	−1.128	0.0280
SPA3G_MOUSE	Q5I2A0	Serine protease inhibitor A3G	1.269	0.0005
P20D1_MOUSE	Q8C165	*N*-fatty-acyl-amino acid synthase/hydrolase PM20D1	1.392	0.0353
PROZ_MOUSE	Q9CQW3	Vitamin K-dependent protein Z	1.545	0.0461
PRDX2_MOUSE	Q61171	Peroxiredoxin-2	1.866	0.0113
APOM_MOUSE	Q9Z1R3	Apolipoprotein M	1.911	0.0182
HA1D_MOUSE	P01902	H-2 class I histocompatibility antigen, K-D alpha chain	2.176	0.0263
HA1W_MOUSE	P03991	H-2 class I histocompatibility antigen, K-W28 alpha chain	2.178	0.0064
CAMP_MOUSE	P51437	Cathelicidin antimicrobial peptide	3.047	0.0327

COOA1_MOUSE, HVM45_MOUSE, MYCD_MOUS, and CP250_MOUSE protein expressions were unique proteins to WD group. Therefore, the respective fold change and *p*-value were not shown.

## Data Availability

The data that support the findings of this study are available from the corresponding author upon reasonable request.

## Supplementary Materials

The following supporting information can be downloaded at: https://www.mdpi.com/article/10.3390/biology11020253/s1, Figure S1: Effects of exercise on body weight, food intake and calorie intake in ApoE^-/-^ mice. The one-way ANOVA followed by Tukey’s post hoc test was used for statistical analysis, and ** *p* < 0.01, *** *p* < 0.001, and **** *p* < 0.0001 represents the significance between ND and WD groups or ND and WD EX groups. ^#^ *p* < 0.05 represents the significance between WD and WD EX groups. n.s. = No significant difference; Table S1: Effects of exercise on the biofunction pathway regulated by proteins between WD and WD EX groups.

## Abbreviation List

Apoe^em1Narl^/Narl, ApoE knockout	Apolipoprotein E knockout mice
CAN	Acetonitrile
CID	Collision-induced dissociation
CVD	Cardiovascular disease
DAB	Diaminobenzidine
DTT	Dithiothreitol
IAA	Iodoacetamide
IOD	Integrated optical density
IPA	Ingenuity pathway analysis
LC-MS/MS	Liquid chromatography-tandem mass spectrometry
ND	Mormal diet
WD EX	WD with 12 weeks exercise intervention
WD	Western diet
