# Supplementary material for: Plasma Proteomic Changes of Atherosclerosis after Exercise in ApoE Knockout Mice
Source: Biology (Basel). 2022 Feb 6;11(2):253. doi: 10.3390/biology11020253 (PMC8869651; doi:10.3390/biology11020253)
Supplement: Supplementary file 1 [file biology-11-00253-s001.zip › biology-1545669-supplementary.pdf]

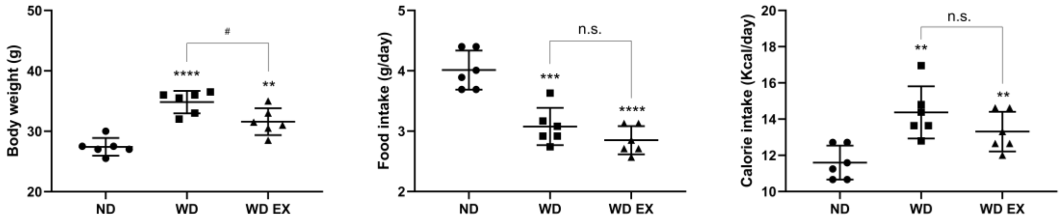

Figure S1. Effects of exercise on body weight, food intake and calorie intake in ApoE$^{-/-}$ mice. The one-way ANOVA followed by Tukey's post hoc test was used for statistical analysis, and ** $p < 0.01$, *** $p < 0.001$, and **** $p < 0.0001$ represents the significance between ND and WD groups or ND and WD EX groups. $^{\#}$ $p < 0.05$ represents the significance between WD and WD EX groups. n.s. = No significant difference.

Table S1. Effects of exercise on the biofunction pathway regulated by proteins between WD and WD EX groups

Ingenuity Canonical Pathways	-log (*p* value)	Activation z-score	Molecules
Cardiovascular Disease, Hematological Disease, Organismal Injury and Abnormalities	15.4763	0.5450	C2, C5, CFB, CFH, CRP, F12, F5, FGG, HABP2, MYOCD, PPIA, PRDX2, PROS1, PROZ, SERPINA10
Inflammatory Disease	13.3585		ACE, AHSG, APOA1, APOM, BHMT, C2, C5, CAMP, CFB, CFH, CRP, F5, HLA-A, HP, HSPA5, HSPA8, IGF1, LAP3, LBP, LCP1, LTF, NRP1, PKM, PPIA, PRDX2, Serpina3g (includes others)
Infectious Diseases	13.3363		AHSG, APOA1, APOM, C5, CFB, CRP, F5, HSPA5, ITIH3, ITIH4, PKM, PPIA
Inflammatory Response	12.7100	-2.1090	ACE, AHSG, APOA1, C5, CAMP, CFH, CRP, F12, FGG, HLA-A, HP, IGF1, LBP, LCP1, LTF, MST1, Orm1 (includes others), PGLYRP2, PPIA, PROS1, Retnlg, SERPINF1
Cell-To-Cell Signaling and Interaction, Hematological System Development and Function, Immune Cell Trafficking, Inflammatory Response	12.5214	-2.2740	ACE, APOA1, C5, CAMP, CFH, CRP, F5, FGG, HLA-A, IGF1, LBP, LTF, Orm1 (includes others), PPIA, PROS1, SERPINF1
Inflammatory Response	12.2161	1.2480	ACE, AMBP, APOA1, APOM, C5, CAMP, CFB, CFH, CRP, F12, F5, HLA-A, HP, HPX, HSPA5, IGF1, LBP, LTF, MST1, PGLYRP2, PKM, PPIA, PRDX2, PROS1, Retnlg, Serpina3g (includes others)
Connective Tissue Disorders, Inflammatory Disease, Inflammatory Response, Organismal Injury and Abnormalities, Skeletal and Muscular Disorders	12.1512	-1.2430	AHSG, APOA1, APOM, BHMT, C2, C5, CFB, CRP, FGG, HLA-A, HP, HSPA5, HSPA8, IGF1, LAP3, LBP, LCP1, LTF, NRP1, PGLYRP2, PKM, PPIA, PRDX2
Inflammatory Response	12.0752	1.5490	ACE, AMBP, APOA1, C5, CAMP, CFB, CFH, CRP, F5, HLA-A, HP, HPX, HSPA5, IGF1, LBP, LTF, MST1, PGLYRP2, PKM, PPIA, PRDX2, PROS1, Retnlg, Serpina3g (includes others)

Table S1. *Cont.*

Ingenuity Canonical Pathways	-log (*p* value)	Activation z-score	Molecules
Metabolic Disease, Organismal Injury and Abnormalities	12.0640		ACE, AHSG, ALDOB, AMBP, APOA1, APOM, BHMT, C2, C5, C8G, C9, CFB, CFH, COL24A1, CRP, HABP2, HLA-A, HP, HSPA5, IGF1, ITIH4, LBP, LCP1, LTF, PKM, PM20D1, PPIA, PRDX2, SERPINF1
Cell-To-Cell Signaling and Interaction, Hematological System Development and Function	11.9318	-2.3830	ACE, C5, CAMP, CFH, CRP, F5, FGG, HLA-A, IGF1, LBP, LTF, Orm1 (includes others), PPIA, PROS1, SERPINF1
Connective Tissue Disorders, Inflammatory Disease, Organismal Injury and Abnormalities, Skeletal and Muscular Disorders	11.2464	-0.9170	AHSG, APOA1, APOM, BHMT, C2, C5, CAMP, CFB, CFH, CRP, FGG, HLA-A, HP, HSPA5, HSPA8, IGF1, LAP3, LBP, LCP1, LTF, NRP1, PGLYRP2, PKM, PPIA, PRDX2
Endocrine System Disorders, Gastrointestinal Disease, Metabolic Disease, Organismal Injury and Abnormalities	11.1765		ACE, AHSG, ALDOB, AMBP, APOA1, APOM, BHMT, C2, C5, C8G, C9, CFB, CFH, COL24A1, CRP, HABP2, HLA-A, HP, HSPA5, IGF1, ITIH4, LCP1, PKM, PM20D1, PPIA, SERPINF1
Cellular Compromise, Inflammatory Response	11.0405		AHSG, APOA1, C5, CAMP, DYNC1H1, F5, FGG, HP, HSPA8, IGF1, ITIH3, ITIH4, LTF, PKM, PPIA, PROS1, PSAP
Connective Tissue Disorders, Immunological Disease, Inflammatory Disease, Inflammatory Response, Organismal Injury and Abnormalities, Skeletal and Muscular Disorders	10.9431		AHSG, APOA1, APOM, BHMT, C2, C5, CFB, CRP, HLA-A, HP, HSPA8, IGF1, LAP3, LBP, LCP1, LTF, NRP1, PPIA, PRDX2
Cell-To-Cell Signaling and Interaction, Hematological System Development and Function, Immune Cell Trafficking, Inflammatory Response	10.9172	-2.2270	ACE, APOA1, C5, CAMP, CFH, CRP, F5, FGG, HLA-A, HPX, IGF1, LBP, LTF, Orm1 (includes others), PGLYRP2, PPIA, PROS1, PSAP, SERPINF1
Immunological Disease, Inflammatory Disease	10.7496		ACE, AHSG, APOA1, APOM, BHMT, C2, C5, CAMP, CFB, CFH, CRP, HLA-A, HP, HPX, HSPA5, HSPA8, IGF1, LAP3, LBP, LCP1, LTF, NRP1, PPIA, PRDX2, PSAP

Table S1. *Cont.*

Ingenuity Canonical Pathways	-log (*p* value)	Activation z-score	Molecules
Inflammatory Response, Organismal Injury and Abnormalities	10.5986	2.1250	ACE, AMBP, APOA1, C5, CAMP, CFB, CFH, CRP, F12, F5, HLA-A, HP, HPX, HSPA5, IGF1, LBP, LTF, MST1, PGLYRP2, PKM, PPIA, PRDX2, PROS1, PSAP, Retnlg, Serpina3g (includes others)
Infectious Diseases	10.5560		ACE, AHSG, APOA1, APOM, C5, CFB, CRP, DYNC1H1, F5, HSPA5, HSPA8, ITIH3, ITIH4, PKM, PPIA
Cellular Compromise, Inflammatory Response	10.4855		AHSG, APOA1, F5, FGG, IGF1, ITIH3, ITIH4, PROS1, PSAP
Infectious Diseases	10.1772	-1.7860	ACE, AHSG, APOA1, APOM, C2, C5, CAMP, CFB, CRP, DYNC1H1, F12, F5, HLA-A, HP, HSPA5, HSPA8, IGF1, ITIH3, ITIH4, LTF, NRP1, PKM, PM20D1, PPIA, PRDX2, PROS1, PSMA7, SERPINA10
Cellular Movement, Immune Cell Trafficking	10.0915	-1.0560	APOA1, C2, C5, CAMP, CFB, CFH, CRP, HLA-A, HP, HSPA5, LBP, LCP1, LTF, MST1, Orm1 (includes others), PGLYRP2, PI16, PPIA, PROS1, Retnlg, SERPINF1
Immunological Disease	10.0625		ACE, AHSG, APOA1, APOM, BHMT, C2, C5, CAMP, CFB, CFH, CRP, F5, HLA-A, HP, HSPA8, IGF1, ITIH4, LAP3, LBP, LCP1, LTF, NRP1, PPIA, PRDX2
Organismal Injury and Abnormalities, Renal and Urological Disease	10.0448	-0.8130	ACE, AHSG, AMBP, C5, CFB, CFH, COLEC11, HPX, IGF1, MST1, PKM
Infectious Diseases	10.0232		ACE, AHSG, APOA1, APOM, C5, CFB, CRP, F5, HSPA5, HSPA8, ITIH3, ITIH4, PKM, PPIA
Cardiovascular Disease, Organismal Injury and Abnormalities	10.0186	-0.4840	ACE, AHSG, APOA1, APOM, C5, CRP, F12, F5, FGG, HABP2, HP, IGF1, PKM, PPIA, PRDX2, PROS1
Cell-To-Cell Signaling and Interaction	9.9469	-1.2210	APOA1, C5, CAMP, CFH, CRP, FGG, LBP, LCP1, LTF, PKM, PRDX2
Free Radical Scavenging	9.9318	0.7730	ACE, APOA1, C5, CAMP, CFH, CRP, HP, IGF1, LTF, PPIA, SERPINF1
Cell-To-Cell Signaling and Interaction, Hematological System Development and Function, Immune Cell Trafficking, Inflammatory Response	9.8996	-1.2430	ACE, APOA1, CAMP, CRP, FGG, HLA-A, IGF1, LBP, LTF, Orm1 (includes others), PROS1, PSAP, SERPINF1

Table S1. *Cont.*

Ingenuity Canonical Pathways	-log (*p* value)	Activation z-score	Molecules
Cell-To-Cell Signaling and Interaction	9.7645	-2.4180	ACE, APOA1, C5, CAMP, CFH, CRP, F5, FGG, HLA-A, HPX, IGF1, LBP, LTF, MST1, Orm1 (includes others), PGLYRP2, PKM, PPIA, PROS1, PSAP, SERPINF1
Cellular Movement	9.6737	-1.7810	AHSG, APOA1, C2, C5, CAMP, CFB, CFH, CRP, F12, HABP2, HLA-A, HP, HSPA5, HSPA8, IGF1, LBP, LCP1, LTF, MST1, MYOCD, NRP1, Orm1 (includes others), PGLYRP2, PI16, PKM, PPIA, PRDX2, PROS1, Retnlg, SERPINF1
Cellular Movement, Hematological System Development and Function, Immune Cell Trafficking	9.5901	-1.2740	APOA1, C2, C5, CAMP, CFB, CFH, CRP, HLA-A, HP, HSPA5, LBP, LCP1, LTF, MST1, PGLYRP2, PI16, PPIA, Retnlg, SERPINF1
Cell-To-Cell Signaling and Interaction	9.5768	-1.2180	APOA1, C5, CAMP, CFH, CRP, F12, F5, FGG, LBP, LCP1, LTF, NRP1, PKM, PRDX2
Organismal Injury and Abnormalities, Respiratory Disease	9.0904	-1.5800	ACE, AHSG, AMBP, C2, C5, CFB, CRP, F12, HABP2, HSPA5, IGF1
Cardiovascular Disease	9.0660	-0.3970	ACE, AHSG, APOA1, APOM, C5, CRP, F12, F5, FGG, HABP2, HP, IGF1, PKM, PPIA, PRDX2
Cell-To-Cell Signaling and Interaction, Hematological System Development and Function, Immune Cell Trafficking, Inflammatory Response	9.0381	-1.1050	ACE, CAMP, CRP, FGG, HLA-A, IGF1, LBP, LTF, Orm1 (includes others), PROS1, SERPINF1
Infectious Diseases	9.0182	-1.9700	ACE, AHSG, APOA1, APOM, C2, C5, CFB, CRP, DYNC1H1, F5, HLA-A, HSPA5, HSPA8, IGF1, ITIH3, ITIH4, LTF, PKM, PM20D1, PPIA, PSMA7
Cardiovascular Disease, Hematological Disease, Hereditary Disorder, Organismal Injury and Abnormalities	8.9245		F5, FGG, HABP2, PROS1
Cell-To-Cell Signaling and Interaction, Hematological System Development and Function, Inflammatory Response	8.9208	-1.4440	APOA1, C5, CAMP, CFH, CRP, FGG, LBP, LCP1, PKM, PRDX2
Cell Death and Survival	8.7375	-0.7850	C5, C8G, C9, CAMP, CFB, CFH, HLA-A, HSPA5, LBP, PPIA, PRDX2

Table S1. *Cont.*

Ingenuity Canonical Pathways	-log (*p* value)	Activation z-score	Molecules
Gastrointestinal Disease, Hepatic System Disease, Organismal Injury and Abnormalities	8.6253	1.3830	ACE, AHSG, ALDOB, AMBP, APOA1, BHMT, C5, C9, CEP250, CFB, CFH, COL24A1, CRP, DYNC1H1, F12, F5, FGG, HABP2, HLA-A, HP, HPX, HSPA5, HSPA8, IGF1, ITIH1, ITIH2, ITIH4, LAP3, LBP, LCP1, LOXL1, MST1, MYOCD, PGLYRP2, PI16, PKM, PM20D1, PPIA, PRDX2, PROS1, PROZ, PSAP, SAA2-SAA4, SERPINA10
Organismal Injury and Abnormalities, Tissue Morphology	8.6198		ACE, APOA1, CAMP, CRP, F12, F5, FGG, HABP2, HLA-A, IGF1, NRP1, PPIA, PRDX2
Organismal Injury and Abnormalities, Renal and Urological Disease	8.4698	-1.1180	AHSG, AMBP, C5, CFB, CFH, COLEC11, IGF1, MST1, PKM
Neurological Disease, Organismal Injury and Abnormalities	8.4609		ACE, APOA1, C5, C9, CFB, CRP, DYNC1H1, F12, F5, HP, HPX, HSPA5, IGF1, LTF, MYOCD, PPIA, PRDX2, PROS1, PSAP
Cell-To-Cell Signaling and Interaction, Hematological System Development and Function, Immune Cell Trafficking	8.4401	-1.4600	APOA1, C5, CAMP, CFH, CRP, FGG, LBP, LCP1, LTF, NRP1, PKM, PRDX2
Organismal Injury and Abnormalities, Respiratory Disease	8.2815	-1.3390	ACE, AHSG, AMBP, C2, C5, CFB, F12, HABP2, HSPA5, IGF1
Cellular Movement	8.2411	-1.1170	APOA1, C2, C5, CAMP, CFB, CFH, CRP, HP, HSPA5, LBP, LCP1, LTF, MST1, PPIA, SERPINF1
Cell Death and Survival, Connective Tissue Disorders, Hematological Disease, Organismal Injury and Abnormalities	8.1931	-0.3390	C5, C8G, CAMP, CFB, CFH, HSPA5, PPIA, PRDX2
Cardiovascular Disease, Organismal Injury and Abnormalities	8.1831	-0.3970	ACE, AHSG, APOA1, APOM, C5, CRP, F12, F5, FGG, HP, IGF1, PKM, PPIA, PRDX2

Table S1. *Cont.*

Ingenuity Canonical Pathways	-log (*p* value)	Activation z-score	Molecules
Cancer, Gastrointestinal Disease, Organismal Injury and Abnormalities	8.1765		ACE, ACTBL2, AHSG, ALDOB, AMBP, APOA1, BHMT, C5, C8G, C9, CEP250, CFB, COL24A1, COLEC11, CRP, DYNC1H1, F12, F5, FGG, HABP2, HLA-A, HP, HPX, HSPA5, HSPA8, IGF1, ITIH1, ITIH2, ITIH4, LAP3, LBP, LOXL1, LTF, MST1, MYOCD, NRP1, PGLYRP2, PI16, PKM, PM20D1, PPIA, PROZ, PSAP, SAA2-SAA4, SERPINA10
Carbohydrate Metabolism	8.1530	-2.3060	APOA1, CAMP, CFH, IGF1, ITIH2, LBP, PSAP
Free Radical Scavenging	8.1415	-1.1230	ACE, APOA1, C5, CAMP, CFB, CFH, CRP, HP, IGF1, ITIH4, LTF, PPIA, PRDX2, SERPINF1
Cell-To-Cell Signaling and Interaction, Hematological System Development and Function	8.1018	-0.8830	C5, CFH, CRP, F12, FGG, IGF1, NRP1, PPIA, PRDX2, SERPINF1
Cardiovascular Disease, Organismal Injury and Abnormalities	8.0311	-0.0860	ACE, AHSG, APOA1, APOM, C5, CRP, F5, FGG, HP, IGF1, PKM, PPIA, PRDX2
Cardiovascular Disease, Organismal Injury and Abnormalities	7.8861	-0.2100	ACE, APOA1, C5, CRP, F12, F5, FGG, HLA-A, IGF1, NRP1, PPIA, PROS1
Cardiovascular System Development and Function, Cellular Development, Cellular Function and Maintenance, Cellular Growth and Proliferation, Organismal Development, Tissue Development	7.8356	-0.0440	CAMP, F12, HABP2, HSPA5, IGF1, NRP1, PKM, PPIA, PROS1
Cardiovascular System Development and Function, Cellular Development, Cellular Function and Maintenance, Cellular Growth and Proliferation, Organismal Development, Tissue Development	7.7328	0.3290	APOA1, CAMP, F12, HABP2, HSPA5, IGF1, NRP1, PKM, PPIA, PROS1, SERPINF1
Cell-To-Cell Signaling and Interaction	7.7122	-1.5340	C5, CFH, CRP, F12, FGG, IGF1, LTF, NRP1, PPIA, PRDX2, SERPINF1

Table S1. *Cont.*

Ingenuity Canonical Pathways	-log (*p* value)	Activation z-score	Molecules
Cardiovascular Disease, Hereditary Disorder, Organismal Injury and Abnormalities	7.5498		ACE, APOA1, CFB, CFH, CRP, F12, F5, FGG, HABP2, IGF1, NRP1, PPIA, PROS1
Hematological System Development and Function	7.4789	-0.9750	C9, CRP, F12, F5, FGG, HP, PPIA, PROS1, PROZ, SERPINF1
Carbohydrate Metabolism	7.4660	-2.1690	APOA1, CAMP, CFH, IGF1, ITIH2, LBP
Cardiovascular Disease, Hereditary Disorder, Organismal Injury and Abnormalities	7.4510		ACE, CFB, CFH, F5, FGG, HABP2, IGF1, NRP1, PROS1
Metabolic Disease, Neurological Disease, Organismal Injury and Abnormalities, Psychological Disorders	7.4473		ACE, APOA1, C5, C9, CFB, CRP, F12, HP, HPX, HSPA5, IGF1, MYOCD, PROS1, PSAP
Endocrine System Disorders, Organismal Injury and Abnormalities	7.3706		AHSG, APOA1, HSPA5, HSPA8, PRDX2
Cellular Function and Maintenance	7.3478	-2.0720	ACE, AMBP, APOA1, C5, CAMP, CFH, COLEC11, CRP, HP, HPX, HSPA5, HSPA8, PKM, PROS1
Cancer, Gastrointestinal Disease, Organismal Injury and Abnormalities	7.3002	0.4380	ACE, AHSG, ALDOB, AMBP, APOA1, BHMT, C5, C9, CEP250, CFB, COL24A1, CRP, DYNC1H1, F12, F5, FGG, HABP2, HLA-A, HP, HPX, HSPA5, HSPA8, IGF1, ITIH1, ITIH2, ITIH4, LAP3, LBP, LCP1, LOXL1, MST1, MYOCD, PGLYRP2, PI16, PKM, PM20D1, PPIA, PRDX2, PROZ, PSAP, SAA2-SAA4, SERPINA10
Cancer, Gastrointestinal Disease, Hepatic System Disease, Organismal Injury and Abnormalities	7.2916		ACE, AHSG, ALDOB, AMBP, APOA1, BHMT, C5, C9, CEP250, CFB, COL24A1, CRP, DYNC1H1, F12, F5, FGG, HABP2, HP, HPX, HSPA5, HSPA8, IGF1, ITIH1, ITIH2, ITIH4, LAP3, LBP, LOXL1, MST1, MYOCD, PGLYRP2, PI16, PKM, PM20D1, PROZ, PSAP, SAA2-SAA4, SERPINA10
Cancer, Gastrointestinal Disease, Hepatic System Disease, Organismal Injury and Abnormalities	7.2526	0.4380	ACE, AHSG, ALDOB, AMBP, APOA1, BHMT, C5, C9, CEP250, CFB, COL24A1, CRP, DYNC1H1, F12, F5, FGG, HABP2, HP, HPX, HSPA5, HSPA8, IGF1, ITIH1, ITIH2, ITIH4, LAP3, LBP, LCP1, LOXL1, MST1, MYOCD, PGLYRP2, PI16, PKM, PM20D1, PPIA, PRDX2, PROZ, PSAP, SAA2-SAA4, SERPINA10

Table S1. *Cont.*

Ingenuity Canonical Pathways	-log (*p* value)	Activation z-score	Molecules
Tissue Development	7.1844	-0.7930	APOA1, C5, CAMP, F12, HABP2, HSPA5, IGF1, ITIH4, LTF, MST1, NRP1, PKM, PPIA, PROS1, SERPINF1
Cancer, Gastrointestinal Disease, Hepatic System Disease, Organismal Injury and Abnormalities	7.1707		ACE, AHSG, ALDOB, AMBP, APOA1, BHMT, C5, C9, CEP250, CFB, COL24A1, CRP, DYNC1H1, F12, F5, FGG, HABP2, HLA-A, HP, HPX, HSPA5, HSPA8, IGF1, ITIH1, ITIH2, ITIH4, LAP3, LBP, LOXL1, MST1, MYOCD, PGLYRP2, PI16, PKM, PM20D1, PROZ, PSAP, SAA2-SAA4, SERPINA10
Protein Synthesis	7.0825		AHSG, APOA1, F5, FGG, IGF1, ITIH2, LTF, SERPINA10
Tissue Development	7.0788	0.1280	APOA1, CAMP, CFH, F12, HABP2, HSPA5, IGF1, MST1, NRP1, PKM, PPIA, PROS1, SERPINF1
Gastrointestinal Disease, Inflammatory Disease, Inflammatory Response, Organismal Injury and Abnormalities	7.0195	0.2770	CAMP, CFB, CRP, HP, LBP, LTF, MST1, PGLYRP2, PKM, PROS1, Retnlg, Serpina3g (includes others)
Lipid Metabolism, Molecular Transport, Small Molecule Biochemistry	6.7825	-1.5610	ACE, AHSG, APOA1, APOM, BHMT, C5, CAMP, CFB, CRP, HP, HSPA5, IGF1, LBP, PKM, PSAP, SAA2-SAA4
Developmental Disorder, Hereditary Disorder, Immunological Disease, Organismal Injury and Abnormalities	6.7033		C2, C5, C9, CFB
Molecular Transport	6.6440		APOA1, C5, CFB, COLEC11, IGF1
Cardiovascular Disease, Hematological Disease, Organismal Injury and Abnormalities	6.6440		F12, F5, FGG, HABP2, PROS1
Cardiovascular System Development and Function	6.6003	-1.1250	ACE, APOA1, C5, CAMP, CFB, CFH, F12, HABP2, HP, HSPA5, IGF1, MYOCD, NRP1, PKM, PPIA, PROS1, PSAP, SERPINF1
Cardiovascular System Development and Function, Organismal Development	6.5513	-1.1410	APOA1, C5, CAMP, CFB, CFH, F12, HABP2, HP, HSPA5, IGF1, MYOCD, NRP1, PKM, PPIA, PROS1, PSAP, SERPINF1

Table S1. *Cont.*

Ingenuity Canonical Pathways	-log (*p* value)	Activation z-score	Molecules
Cardiovascular Disease, Cellular Compromise, Organismal Injury and Abnormalities	6.5361	2.0000	CRP, HP, HPX, SERPINF1
Cell-To-Cell Signaling and Interaction, Hematological System Development and Function, Inflammatory Response	6.5017	-0.2620	CFH, CRP, F12, FGG, IGF1, PPIA, PRDX2, SERPINF1
Hematological System Development and Function, Organismal Functions	6.4855	-0.6470	C9, CRP, F12, F5, FGG, PPIA, PROS1, PROZ, SERPINF1
Cellular Compromise, Inflammatory Response	6.4802		AHSG, C5, CAMP, DYNC1H1, HP, HSPA8, LTF, PKM, PPIA, PSAP
Cellular Function and Maintenance	6.4318	-2.2340	ACE, AHSG, APOA1, C5, CAMP, CFH, CRP, HP, HSPA5, HSPA8, PKM, PROS1
Cellular Movement	6.4023	0.5690	APOA1, C2, C5, CFB, CFH, HLA-A, HSPA5, LBP, LTF, NRP1, PI16, PPIA
Organismal Survival	6.3726	0.1210	APOA1, C5, CAMP, CFB, CFH, CRP, F12, HLA-A, HP, IGF1, LBP, LTF, NRP1, PKM, SERPINA10
Cellular Movement, Hematological System Development and Function, Immune Cell Trafficking, Inflammatory Response	6.3468	-1.2980	APOA1, C2, C5, CAMP, CFB, CFH, CRP, HP, HSPA5, LBP, MST1, PPIA, SERPINF1
Metabolic Disease, Organismal Injury and Abnormalities	6.3420	2.0000	ACE, APOA1, CRP, HSPA5, LBP, LCP1, PPIA, PSAP, SERPINF1
Cell Death and Survival, Organismal Injury and Abnormalities, Renal and Urological Disease	6.3391	0.6920	C5, CFB, IGF1, MST1, PKM
Dermatological Diseases and Conditions, Organismal Injury and Abnormalities	6.3206		C5, CAMP, CRP, HLA-A, NRP1, PPIA, PSAP
Cardiovascular Disease, Hematological Disease, Hereditary Disorder, Organismal Injury and Abnormalities	6.2984		F5, HABP2, PROS1

Table S1. *Cont.*

Ingenuity Canonical Pathways	-log (*p* value)	Activation z-score	Molecules
Cell Death and Survival	6.2984		ACE, CAMP, LTF
Carbohydrate Metabolism, Lipid Metabolism, Molecular Transport, Small Molecule Biochemistry	6.2984		CAMP, CFB, LBP
Endocrine System Disorders, Gastrointestinal Disease, Inflammatory Disease, Inflammatory Response, Organismal Injury and Abnormalities	6.2907		APOA1, F5, HSPA5, IGF1, PKM, PPIA, PRDX2
Cellular Movement, Hematological System Development and Function, Immune Cell Trafficking, Inflammatory Response	6.2790	-1.4600	APOA1, C5, CAMP, HP, MST1, PPIA
Organismal Injury and Abnormalities	6.2668	1.5200	ACE, C5, CRP, F12, F5, FGG, NRP1, PGLYRP2, PPIA, PROS1, PROZ
Cell Death and Survival	6.2652	-0.8820	ACE, C9, CAMP, CFH, HLA-A, HSPA5, LTF, SERPINF1
Cardiovascular System Development and Function, Organismal Development	6.2336	-0.9550	APOA1, C5, CAMP, CFB, F12, HABP2, HSPA5, IGF1, MYOCD, NRP1, PKM, PPIA, PROS1, PSAP, SERPINF1
Hematological Disease	6.1688	-1.0670	C5, CFB, F12, F5, FGG, HSPA5, LTF, PPIA, PROS1
Humoral Immune Response, Inflammatory Response	6.1543		C2, CFB, CFH, COLEC11, CRP, F12
Cellular Movement	6.1124	-1.5810	APOA1, C5, CAMP, HP, IGF1, LBP, LCP1, MST1, NRP1, PPIA, Retnlg, SERPINF1
Cell-To-Cell Signaling and Interaction, Hematological System Development and Function, Inflammatory Response	6.0429	-1.0000	CRP, FGG, LBP, LCP1, PRDX2
Metabolic Disease, Organismal Injury and Abnormalities	6.0255	2.0000	ACE, APOA1, CRP, HSPA5, LBP, LCP1, PPIA, PROS1, PSAP, SERPINF1
Cell Death and Survival	6.0250	2.2220	ACE, APOA1, C5, C9, CAMP, CFB, CFH, CRP, DYNC1H1, F5, HLA-A, HSPA5, HSPA8, IGF1, ITIH4, LBP, LTF, MST1, MYOCD, NRP1, PKM, PM20D1, PPIA, PRDX2, PROS1, PSAP, PSMA7, SERPINF1

Table S1. *Cont.*

Ingenuity Canonical Pathways	-log (*p* value)	Activation z-score	Molecules
Inflammatory Response, Respiratory Disease	6.0097		ACE, AMBP, APOA1, C5, CAMP, CRP, HLA-A, HSPA5, IGF1, LBP, LTF, PPIA
Cellular Movement, Hematological System Development and Function, Immune Cell Trafficking	6.0083	0.0980	APOA1, C2, C5, CFB, CFH, HLA-A, HSPA5, LBP, LTF, PI16, PPIA
Cell-To-Cell Signaling and Interaction	6.0044	-1.3870	APOA1, C5, CFH, CRP, LCP1, LTF, PRDX2
Cancer, Gastrointestinal Disease, Hepatic System Disease, Organismal Injury and Abnormalities	5.9914		ALDOB, BHMT, HSPA5, HSPA8
Cardiovascular System Development and Function	5.9914	0.0140	C5, CAMP, CFB, IGF1, LTF, NRP1, SERPINF1
Hereditary Disorder, Organismal Injury and Abnormalities, Psychological Disorders	5.9914		ACE, C5, C9, CRP, DYNC1H1, FGG, HP, HSPA5, HSPA8, IGF1, ITIH3, ITIH4, PKM, PPIA, PRDX2, PSAP
Cardiovascular System Development and Function, Cellular Movement	5.9136	-0.2980	APOA1, C5, CAMP, CRP, HSPA5, IGF1, NRP1, PKM, PROS1, SERPINF1
Cellular Function and Maintenance	5.9136	-1.8990	ACE, APOA1, C5, CAMP, CFH, CRP, HP, HSPA5, PKM, PROS1
Infectious Diseases	5.8794		C5, F12, F5, PROS1
Cardiovascular Disease, Hematological Disease, Organismal Injury and Abnormalities, Renal and Urological Disease	5.8794		C2, C5, CFB, CFH
Cellular Movement, Hematological System Development and Function, Immune Cell Trafficking, Inflammatory Response	5.7959	-1.6980	APOA1, C5, CAMP, HP, MST1, PPIA, SERPINF1
Hematological Disease	5.7721		F12, F5, FGG, PPIA
Hematological Disease, Metabolic Disease, Organismal Injury and Abnormalities	5.7696		ACE, APOA1, CRP, LBP, LCP1, PPIA, SERPINF1

Table S1. *Cont.*

Ingenuity Canonical Pathways	-log (*p* value)	Activation z-score	Molecules
Dermatological Diseases and Conditions, Organismal Injury and Abnormalities	5.7645		C5, CAMP, CRP, HSPA8, NRP1, PKM, PPIA, PSAP
Cellular Movement, Hematological System Development and Function, Immune Cell Trafficking	5.7399	0.0250	APOA1, C2, C5, CAMP, CFB, CFH, CRP, LBP, LTF, PPIA
Cardiovascular Disease, Cell Death and Survival, Connective Tissue Disorders, Hematological Disease, Organismal Injury and Abnormalities	5.7122		C5, CFB, CFH, HSPA5, PPIA, PRDX2
Respiratory Disease	5.6925		ACE, AHSG, C5, CAMP, F12, HABP2, HP, HSPA5, LTF
Cardiovascular Disease, Hematological Disease, Organismal Injury and Abnormalities	5.6737		C5, CFB, CFH, PPIA
Carbohydrate Metabolism, Lipid Metabolism, Small Molecule Biochemistry	5.6737	-1.9820	C5, HSPA5, IGF1, PRDX2
Organismal Development, Organismal Injury and Abnormalities	5.6737		ACE, AMBP, APOA1, BHMT, CFH, COLEC11, HP, HPX, IGF1, LOXL1, MST1, MYOCD, PGLYRP2, PRDX2, PSAP
Cellular Function and Maintenance	5.6696	1.0300	ACE, APOA1, C5, CAMP, CFH, CRP, F5, HLA-A, HPX, HSPA5, HSPA8, IGF1, ITIH3, LTF, MYOCD, PGLYRP2, PKM, PPIA, PRDX2, PSAP, SERPINF1
Hematological Disease, Hereditary Disorder, Organismal Injury and Abnormalities	5.6271		F5, HABP2, PROS1
Cellular Movement, Hematological System Development and Function, Immune Cell Trafficking, Inflammatory Response	5.6271	-0.8490	APOA1, CAMP, LCP1, PPIA
Cellular Function and Maintenance, Hematological System Development and Function	5.6253	-1.6670	ACE, APOA1, C5, CAMP, CFH, CRP, PROS1

Table S1. *Cont.*

Ingenuity Canonical Pathways	-log (*p* value)	Activation z-score	Molecules
Cardiovascular Disease, Neurological Disease, Organismal Injury and Abnormalities	5.6216		ACE, APOA1, CFH, CRP, F5, IGF1, NRP1, PROS1
Cellular Movement, Hematological System Development and Function, Immune Cell Trafficking, Inflammatory Response	5.6126	-1.5850	APOA1, C5, CAMP, HP, LCP1, MST1, PPIA
Cellular Movement	5.5498	-1.6070	C5, HABP2, HSPA5, IGF1, NRP1, PKM, PPIA, PROS1
Cell-To-Cell Signaling and Interaction	5.5421	-0.9950	CAMP, CFH, HLA-A, HSPA5, IGF1, LTF, NRP1, PKM, PROS1, SERPINF1
Cellular Development, Cellular Growth and Proliferation, Hematological System Development and Function, Hematopoiesis, Lymphoid Tissue Structure and Development, Tissue Development	5.5258	0.3890	CAMP, CRP, F5, HLA-A, HP, HPX, IGF1, LCP1, LTF, NRP1, PGLYRP2, PKM, PRDX2, PSAP
Neurological Disease, Skeletal and Muscular Disorders	5.5045		C5, C9, CFB, CRP, FGG, HP, HSPA5, HSPA8, IGF1, ITIH3, ITIH4, LTF, PKM, PPIA, PRDX2, PSAP
Cellular Function and Maintenance	5.4989	-1.6670	ACE, APOA1, C5, CAMP, CFH, CRP, PROS1
Cellular Movement, Hematological System Development and Function, Immune Cell Trafficking, Inflammatory Response	5.4776	-0.5200	APOA1, C2, C5, CAMP, CFB, CFH, CRP, LBP, PPIA
Cell-To-Cell Signaling and Interaction, Hematological System Development and Function, Inflammatory Response	5.4365	-0.9370	C5, CAMP, CFH, CRP, PROS1
Dermatological Diseases and Conditions, Organismal Injury and Abnormalities	5.3936		C5, CAMP, CRP, HSPA5, HSPA8, LTF, NRP1, PKM, PM20D1, PPIA, PSAP
Cell-To-Cell Signaling and Interaction, Hematological System Development and Function, Immune Cell Trafficking	5.3526	-0.8970	APOA1, C5, CFH, LBP, LCP1, LTF

Table S1. *Cont.*

Ingenuity Canonical Pathways	-log (*p* value)	Activation z-score	Molecules
Cardiovascular System Development and Function, Cellular Movement	5.3307	-0.5740	APOA1, C5, CRP, HSPA5, IGF1, NRP1, PKM, PROS1, SERPINF1
Lipid Metabolism, Small Molecule Biochemistry	5.3261	-1.2360	APOA1, CAMP, IGF1, LBP, PSAP
Cell-To-Cell Signaling and Interaction, Cellular Function and Maintenance, Inflammatory Response	5.3098	-1.6260	ACE, AHSG, APOA1, CAMP, CFH, CRP, HSPA8, PKM, PROS1
Protein Synthesis	5.2832	-0.5150	APOA1, APOM, BHMT, CRP, FGG
Cellular Movement, Hematological System Development and Function, Immune Cell Trafficking, Inflammatory Response	5.2765	-1.5120	APOA1, C5, CAMP, HP, LBP, LCP1, MST1, PPIA, Retnlg
Cardiovascular System Development and Function, Organismal Development	5.2676	-0.1330	C5, CAMP, CFB, IGF1, NRP1, SERPINF1
Neurological Disease	5.2644		C5, C9, CRP, DYNC1H1, FGG, HP, HSPA5, HSPA8, IGF1, ITIH3, ITIH4, LTF, NRP1, PKM, PPIA, PRDX2, PSAP
Cancer, Organismal Injury and Abnormalities	5.2557	0.2450	ACE, ACTBL2, AHSG, ALDOB, AMBP, APOA1, BHMT, C2, C5, C8G, C9, CAMP, CEP250, CFB, CFH, COL24A1, COLEC11, CRP, DYNC1H1, F12, F5, FGG, HABP2, HLA-A, HP, HPX, HSPA5, HSPA8, IGF1, ITIH1, ITIH2, ITIH3, ITIH4, LAP3, LCP1, LOXL1, LTF, MST1, MYOCD, NRP1, PGLYRP2, PI16, PKM, PM20D1, PPIA, PRDX2, PROS1, PROZ, PSMA7, SAA2-SAA4, SERPINF1
Molecular Transport	5.2480	-1.9580	ACE, APOA1, CAMP, CRP, FGG, HSPA5, HSPA8, IGF1, PKM, PPIA, PSAP
Cancer, Gastrointestinal Disease, Organismal Injury and Abnormalities	5.2381		ACE, ALDOB, AMBP, APOA1, BHMT, C5, C9, CEP250, CFB, COL24A1, CRP, DYNC1H1, F12, F5, FGG, HABP2, HLA-A, HP, HPX, HSPA5, HSPA8, IGF1, ITIH1, ITIH2, ITIH4, LAP3, LOXL1, MYOCD, PI16, PKM, PM20D1, PROZ, PSAP, SAA2-SAA4, SERPINA10

Table S1. *Cont.*

Ingenuity Canonical Pathways	-log (*p* value)	Activation z-score	Molecules
Neurological Disease, Organismal Injury and Abnormalities, Psychological Disorders	5.2218		C9, CRP, FGG, HP, HSPA5, HSPA8, IGF1, ITIH3, ITIH4, LTF, PKM, PPIA, PRDX2, PSAP
Cell-To-Cell Signaling and Interaction, Hematological System Development and Function	5.2218	-0.8160	APOA1, CRP, FGG, LBP, LCP1, NRP1, PRDX2
Hereditary Disorder, Ophthalmic Disease, Organismal Injury and Abnormalities	5.2182		C2, CFB
Organismal Injury and Abnormalities, Renal and Urological Disease	5.2182		CFB, CRP
Cardiovascular Disease, Hematological Disease, Organismal Injury and Abnormalities	5.2132		AMBP, C5, CFB, CFH, CRP, HPX, HSPA5, IGF1, PPIA, PRDX2
Cell-To-Cell Signaling and Interaction	5.1904		CFH, HABP2, LTF
Cancer, Organismal Injury and Abnormalities, Reproductive System Disease	5.1772		ACE, ACTBL2, ALDOB, APOA1, C2, C5, C9, CAMP, CEP250, CFB, CFH, COL24A1, COLEC11, CRP, DYNC1H1, F5, FGG, HABP2, HLA-A, HP, HPX, HSPA5, IGF1, ITIH1, ITIH2, ITIH3, ITIH4, LAP3, LBP, LCP1, LOXL1, LTF, MST1, MYOCD, NRP1, PI16, PKM, PM20D1, PPIA, PROS1, PSMA7, SAA2-SAA4, SERPINF1
Cell Death and Survival	5.1421	1.9640	HSPA5, IGF1, PROS1, SERPINF1
Cell-To-Cell Signaling and Interaction	5.1319	-1.4800	ACE, APOA1, C5, CAMP, CFH, CRP, PROS1
Hereditary Disorder, Neurological Disease, Organismal Injury and Abnormalities, Psychological Disorders, Skeletal and Muscular Disorders	5.1296		C9, CRP, FGG, HP, HSPA5, HSPA8, IGF1, ITIH3, ITIH4, PKM, PPIA, PRDX2
Carbohydrate Metabolism	5.1079	-0.2100	AMBP, APOA1, BHMT, C5, CAMP, CFB, HSPA5, IGF1, LBP, LTF, PKM

Table S1. *Cont.*

Ingenuity Canonical Pathways	-log (*p* value)	Activation z-score	Molecules
Carbohydrate Metabolism, Lipid Metabolism, Small Molecule Biochemistry	5.1007		APOA1, CAMP, LBP
Cell-To-Cell Signaling and Interaction, Hematological System Development and Function, Immune Cell Trafficking, Inflammatory Response	5.0804	-1.7090	APOA1, C5, CFH, CRP, LCP1, PRDX2
Cell Death and Survival, Organismal Injury and Abnormalities, Renal and Urological Disease	5.0731	0.2180	C5, CFB, IGF1, MST1
Protein Synthesis	5.0565	-1.7010	ACE, AHSG, AMBP, APOA1, APOM, C5, F12, F5, FGG, HABP2, HSPA5, HSPA8, IGF1, ITIH2, LTF, SERPINA10
Cardiovascular Disease, Neurological Disease, Organismal Injury and Abnormalities	5.0448		APOA1, F5, IGF1, NRP1, PROS1
Cardiovascular Disease, Organismal Injury and Abnormalities	5.0177		ACE, AHSG, APOA1, CFH, CRP, F5, IGF1, LCP1, NRP1, PROS1
Cell Death and Survival	5.0173		C9, CFB, CFH
Endocrine System Disorders, Metabolic Disease, Organismal Injury and Abnormalities	5.0173		ACE, APOA1, CRP, LBP, LTF, PRDX2
Organismal Injury and Abnormalities, Respiratory Disease	5.0173		ACE, AHSG, C5, F12, HABP2, HSPA5
Endocrine System Disorders, Gastrointestinal Disease, Inflammatory Disease, Inflammatory Response, Organismal Injury and Abnormalities	5.0074		APOA1, HSPA5, PKM, PRDX2

Table S1. *Cont.*

Ingenuity Canonical Pathways	-log (*p* value)	Activation z-score	Molecules
Cancer, Organismal Injury and Abnormalities	4.9957		ACE, ACTBL2, APOA1, C2, C5, C9, CEP250, CFB, CFH, COL24A1, CRP, DYNC1H1, F5, HLA-A, HP, HPX, HSPA5, IGF1, ITIH3, LBP, LCP1, LOXL1, LTF, MST1, MYOCD, NRP1, PI16, PKM, PM20D1, PPIA, PSMA7, SERPINF1
Cellular Function and Maintenance	4.9957	0.8160	C5, CFH, CRP, F5, HLA-A, HPX, IGF1, PGLYRP2, PKM, PRDX2, PSAP
Cardiovascular Disease, Hematological Disease, Metabolic Disease, Organismal Injury and Abnormalities	4.9914		APOA1, CRP, LBP, LCP1, PPIA, SERPINF1
Cardiovascular Disease, Organismal Injury and Abnormalities	4.9706	1.3420	CRP, HP, HPX, PI16, SERPINF1
Cancer, Gastrointestinal Disease, Hepatic System Disease, Organismal Injury and Abnormalities	4.9547		ALDOB, BHMT, HSPA5, HSPA8, PKM
Cardiovascular Disease, Cell Death and Survival, Connective Tissue Disorders, Hematological Disease, Organismal Injury and Abnormalities, Renal and Urological Disease	4.9393		C5, CFB, CFH
Embryonic Development, Organismal Development	4.9355		ACE, APOA1, C5, CFH, HP, IGF1, LBP, MST1, MYOCD, PKM, PSAP
Cardiovascular System Development and Function, Tissue Morphology	4.9136	-1.1140	APOA1, CAMP, CRP, IGF1
Free Radical Scavenging	4.8996	-2.2360	ACE, C5, CAMP, CFB, CRP, IGF1, LTF, PPIA, PRDX2
Carbohydrate Metabolism	4.8962	-0.9260	ACE, AHSG, APOA1, C5, CAMP, HSPA5, IGF1, PKM

Table S1. *Cont.*

Ingenuity Canonical Pathways	-log (*p* value)	Activation z-score	Molecules
Cellular Development, Cellular Growth and Proliferation, Hematological System Development and Function, Hematopoiesis, Lymphoid Tissue Structure and Development, Tissue Development	4.8962	-0.3630	CAMP, CRP, F5, HLA-A, HPX, IGF1, LCP1, LTF, PGLYRP2, PKM, PRDX2, PSAP
Free Radical Scavenging, Molecular Transport	4.8827		C5, CAMP, IGF1, PRDX2
Embryonic Development, Organismal Development	4.8729		ACE, APOA1, C5, CFH, F5, HP, HSPA5, IGF1, LBP, LOXL1, MST1, MYOCD, NRP1, PKM, PRDX2, PSAP
Inflammatory Response	4.8665		AHSG, CRP, LBP
Cell Death and Survival, Organismal Injury and Abnormalities, Renal and Urological Disease	4.8477	0.6920	C5, CFB, IGF1, MST1, PKM
Cell-To-Cell Signaling and Interaction, Inflammatory Response	4.8041	-1.4740	ACE, APOA1, C5, CAMP, CFH, CRP, PROS1
Cancer, Organismal Injury and Abnormalities, Reproductive System Disease	4.7986		ACTBL2, APOA1, C2, C5, C9, CEP250, CFB, CFH, COL24A1, CRP, DYNC1H1, F5, HLA-A, HP, HPX, HSPA5, IGF1, ITIH3, LBP, LCP1, LOXL1, LTF, MST1, MYOCD, NRP1, PI16, PKM, PM20D1, PPIA, PSMA7, SERPINF1
Dermatological Diseases and Conditions, Hematological Disease, Immunological Disease, Organismal Injury and Abnormalities	4.7959		C5, HLA-A, PPIA
Immunological Disease	4.7852		C2, C5, C9, CFB, CFH, FGG, IGF1, LTF
Neurological Disease	4.7721		APOA1, C9, CRP, FGG, HP, HSPA5, HSPA8, IGF1, ITIH3, ITIH4, PKM, PPIA, PRDX2
Cardiovascular System Development and Function, Organismal Development, Visual System Development and Function	4.7645	-0.6890	C5, CFB, IGF1, NRP1, SERPINF1

Table S1. *Cont.*

Ingenuity Canonical Pathways	-log (*p* value)	Activation z-score	Molecules
Cell Death and Survival, Organismal Injury and Abnormalities	4.7496	0.2010	ACE, CAMP, CRP, HSPA5, IGF1, ITIH4, MST1, PKM, PPIA, PRDX2, SERPINF1
Organismal Injury and Abnormalities, Renal and Urological Disease	4.7423		CFB, CFH
Cancer, Organismal Injury and Abnormalities	4.7423	-0.1920	ACE, ACTBL2, AHSG, ALDOB, AMBP, APOA1, BHMT, C2, C5, C8G, C9, CEP250, CFB, CFH, COL24A1, COLEC11, CRP, DYNC1H1, F12, F5, FGG, HABP2, HLA-A, HP, HPX, HSPA5, HSPA8, IGF1, ITIH1, ITIH2, ITIH3, ITIH4, LAP3, LCP1, LOXL1, LTF, MST1, MYOCD, NRP1, PGLYRP2, PI16, PKM, PM20D1, PPIA, PRDX2, PROS1, PROZ, PSMA7, SAA2-SAA4
Cellular Movement, Hematological System Development and Function, Immune Cell Trafficking	4.7399	-1.7910	APOA1, C5, CAMP, HLA-A, HP, LCP1, MST1, PGLYRP2, PPIA, SERPINF1
Cell-To-Cell Signaling and Interaction, Cellular Function and Maintenance, Hematological System Development and Function, Inflammatory Response	4.7399		CAMP, CFH, CRP, PROS1
Inflammatory Response	4.7352	-1.8640	ACE, AHSG, APOA1, C5, CAMP, CFH, CRP, HSPA8, LTF, PKM, PROS1
Cardiovascular Disease, Organismal Injury and Abnormalities, Skeletal and Muscular Disorders	4.7328		ACE, APOA1, C5, CRP, PPIA
Cellular Movement, Hematological System Development and Function, Immune Cell Trafficking, Inflammatory Response	4.7305	-0.5070	APOA1, C2, C5, CFB, CFH, HSPA5, LBP, PPIA
Dermatological Diseases and Conditions, Organismal Injury and Abnormalities	4.7282		C5, HLA-A, PPIA

Table S1. *Cont.*

Ingenuity Canonical Pathways	-log (*p* value)	Activation z-score	Molecules
Cell-To-Cell Signaling and Interaction, Cellular Function and Maintenance, Hematological System Development and Function	4.7235	-1.1450	ACE, APOA1, CAMP, CFH, CRP, PROS1
Ophthalmic Disease, Organismal Injury and Abnormalities	4.7122		C2, C9, CFB, CFH
Cell-To-Cell Signaling and Interaction, Cellular Function and Maintenance, Inflammatory Response	4.7033	-1.1450	ACE, APOA1, CAMP, CFH, CRP, PROS1
Cellular Movement, Hematological System Development and Function, Immune Cell Trafficking	4.7011	-0.5640	APOA1, C2, C5, CFB, CFH, LBP, PPIA
Respiratory Disease	4.7011		ACE, C5, CAMP, F12, HABP2, HP, LTF
Gastrointestinal Disease, Organismal Injury and Abnormalities	4.6861	-1.1310	C2, C5, CFB, LTF
Cardiovascular Disease, Organismal Injury and Abnormalities	4.6799		ACE, APOA1, C5, CRP, F12, PPIA
Antimicrobial Response, Inflammatory Response	4.6778		C5, CAMP, COLEC11, HLA-A, HSPA8, LTF, PGLYRP2, PPIA
Organismal Development	4.6716		ACE, AMBP, APOA1, BHMT, C5, CFB, CFH, COLEC11, HP, HPX, IGF1, LOXL1, MST1, MYOCD, NRP1, PGLYRP2, PPIA, PRDX2, PSAP
Organismal Survival	4.6655	3.4150	ACE, AMBP, APOA1, C5, CAMP, CFB, CFH, CRP, DYNC1H1, F5, FGG, HP, HSPA5, IGF1, LCP1, MYOCD, NRP1, PGLYRP2, PKM, PPIA, PROS1, PROZ, PSAP, SERPINA10
Cardiovascular Disease, Organismal Injury and Abnormalities	4.6478		APOA1, CRP, F5, FGG, IGF1, PPIA
Cell-To-Cell Signaling and Interaction, Inflammatory Response	4.6459	-1.3480	ACE, APOA1, C5, CAMP, CFH, CRP, LTF, PROS1

Table S1. *Cont.*

Ingenuity Canonical Pathways	-log (*p* value)	Activation z-score	Molecules
Organismal Injury and Abnormalities, Renal and Urological Disease	4.6289		ACE, C5, CFB, CRP, F5, MST1, PPIA
Gastrointestinal Disease, Inflammatory Disease, Inflammatory Response, Organismal Injury and Abnormalities	4.6271		CAMP, CFB, HP, LBP, Retnlg, Serpina3g (includes others)
Dermatological Diseases and Conditions, Gastrointestinal Disease, Immunological Disease, Organismal Injury and Abnormalities	4.6055		C5, HLA-A, PPIA
Infectious Diseases	4.5969		C5, CAMP, CRP, F5, HLA-A, LBP, LTF
Cancer, Organismal Injury and Abnormalities	4.5901		ACE, ACTBL2, ALDOB, AMBP, APOA1, C2, C5, C8G, C9, CAMP, CEP250, CFB, CFH, COL24A1, COLEC11, CRP, DYNC1H1, F5, FGG, HABP2, HLA-A, HP, HPX, HSPA5, HSPA8, IGF1, ITIH1, ITIH2, ITIH3, ITIH4, LAP3, LBP, LCP1, LOXL1, LTF, MST1, MYOCD, NRP1, PI16, PKM, PM20D1, PPIA, PRDX2, PROS1, PSAP, PSMA7, SAA2-SAA4, SERPINF1
Endocrine System Disorders, Gastrointestinal Disease, Immunological Disease, Metabolic Disease, Organismal Injury and Abnormalities	4.5784		APOM, CFB, CFH, CRP, HLA-A, IGF1, ITIH4, LCP1, PPIA
Renal and Urological System Development and Function	4.5751		ACE, C5, CFH, COLEC11, HP, IGF1, MYOCD, PSAP
Cancer, Organismal Injury and Abnormalities	4.5719	-0.0540	ACE, ACTBL2, AHSG, ALDOB, AMBP, APOA1, BHMT, C2, C5, C8G, C9, CEP250, CFB, CFH, COL24A1, COLEC11, CRP, DYNC1H1, F12, F5, FGG, HABP2, HLA-A, HP, HPX, HSPA5, HSPA8, IGF1, ITIH1, ITIH2, ITIH3, ITIH4, LAP3, LCP1, LOXL1, LTF, MST1, MYOCD, NRP1, PGLYRP2, PI16, PKM, PM20D1, PPIA, PRDX2, PROS1, PROZ, PSMA7, SAA2-SAA4

Table S1. *Cont.*

Ingenuity Canonical Pathways	-log (*p* value)	Activation z-score	Molecules
Dermatological Diseases and Conditions, Organismal Injury and Abnormalities	4.5331		C5, F5, PPIA, PROS1
Cardiovascular Disease, Hematological Disease, Metabolic Disease, Organismal Injury and Abnormalities	4.5100		APOA1, CRP, LCP1, SERPINF1
Cell-To-Cell Signaling and Interaction, Cellular Function and Maintenance, Inflammatory Response	4.5100	-0.6510	ACE, CAMP, CFH, CRP
Cellular Assembly and Organization, Inflammatory Response	4.4949		ACE, C5, HPX
Cell-To-Cell Signaling and Interaction, Hematological System Development and Function, Immune Cell Trafficking	4.4921	-0.8000	APOA1, C5, CFH, LCP1, LTF
Cardiovascular Disease, Organismal Injury and Abnormalities, Tissue Morphology	4.4750		APOA1, CRP, F12, FGG, HLA-A, PPIA
Organismal Injury and Abnormalities, Renal and Urological Disease	4.4750	0.1520	ACE, CFB, CFH, CRP, MST1, PKM
Cardiovascular Disease, Organismal Injury and Abnormalities	4.4647		ACE, APOA1, C5, CRP, F5, PPIA
Cellular Movement	4.4634	-0.3750	APOA1, C2, C5, CFB, CFH, HSPA5, LBP, PPIA
Protein Synthesis	4.4622	-0.7830	APOA1, APOM, BHMT, CRP
Cardiovascular Disease, Hematological Disease, Organismal Injury and Abnormalities	4.4622		F5, HABP2, PROS1, SERPINA10

Table S1. *Cont.*

Ingenuity Canonical Pathways	-log (*p* value)	Activation z-score	Molecules
Cancer, Organismal Injury and Abnormalities	4.4597		ACE, ACTBL2, AHSG, ALDOB, AMBP, APOA1, BHMT, C2, C5, C8G, C9, CEP250, CFB, CFH, COL24A1, COLEC11, CRP, DYNC1H1, F12, F5, FGG, HABP2, HLA-A, HP, HPX, HSPA5, HSPA8, IGF1, ITIH1, ITIH2, ITIH3, ITIH4, LAP3, LBP, LCP1, LOXL1, LTF, MST1, MYOCD, NRP1, PGLYRP2, PI16, PKM, PM20D1, PPIA, PRDX2, PROS1, PROZ, PSAP, PSMA7, SAA2-SAA4, SERPINF1
Hematological System Development and Function	4.4413		IGF1, LBP
Endocrine System Disorders, Gastrointestinal Disease, Immunological Disease, Inflammatory Disease, Inflammatory Response, Organismal Injury and Abnormalities	4.4413		APOA1, PKM, PRDX2
Cellular Movement, Hair and Skin Development and Function	4.4413		APOA1, CAMP, PPIA
Cardiovascular System Development and Function, Cell-To-Cell Signaling and Interaction	4.4168		C5, CAMP, LBP, PPIA
Inflammatory Disease, Inflammatory Response, Organismal Injury and Abnormalities, Renal and Urological Disease	4.4045	1.4670	ACE, AMBP, C5, CFB, CFH, CRP, IGF1, PPIA
Organismal Injury and Abnormalities, Reproductive System Disease	4.3947		C5, F5, FGG, PPIA
Cardiovascular System Development and Function, Cellular Movement	4.3605	-1.7390	C5, HSPA5, IGF1, NRP1, PKM, PROS1

Table S1. *Cont.*

Ingenuity Canonical Pathways	-log (*p* value)	Activation z-score	Molecules
Cancer, Organismal Injury and Abnormalities	4.3556	0.2360	ACE, ACTBL2, AHSG, ALDOB, AMBP, APOA1, BHMT, C2, C5, C8G, C9, CAMP, CEP250, CFB, CFH, COL24A1, COLEC11, CRP, DYNC1H1, F12, F5, FGG, HABP2, HLA-A, HP, HPX, HSPA5, HSPA8, IGF1, ITIH1, ITIH2, ITIH3, ITIH4, LAP3, LCP1, LOXL1, LTF, MST1, MYOCD, NRP1, PGLYRP2, PI16, PKM, PM20D1, PPIA, PRDX2, PROS1, PROZ, PSAP, PSMA7, SAA2-SAA4, SERPINA10, SERPINF1
Cancer, Gastrointestinal Disease, Hepatic System Disease, Organismal Injury and Abnormalities	4.3507		ACE, ALDOB, APOA1, BHMT, C5, C9, CEP250, CFB, COL24A1, DYNC1H1, F12, F5, FGG, HABP2, HP, HPX, HSPA5, HSPA8, IGF1, ITIH1, ITIH2, ITIH4, LAP3, LOXL1, MYOCD, PI16, PKM, PM20D1, PROZ, PSAP, SAA2-SAA4, SERPINA10
Cell-To-Cell Signaling and Interaction	4.3429		C5, CAMP, IGF1
Gastrointestinal Disease, Organismal Injury and Abnormalities	4.3429		C2, C5, CFB
Cancer, Gastrointestinal Disease, Hepatic System Disease, Organismal Injury and Abnormalities	4.3429		HSPA5, HSPA8, LAP3
Cell-To-Cell Signaling and Interaction, Hematological System Development and Function	4.3420	-0.4470	APOA1, CRP, FGG, LBP, LCP1, PRDX2
Cancer, Organismal Injury and Abnormalities	4.3401	-0.1520	ACE, ACTBL2, AHSG, ALDOB, AMBP, APOA1, BHMT, C2, C5, C8G, C9, CEP250, CFB, CFH, COL24A1, COLEC11, CRP, DYNC1H1, F12, F5, FGG, HABP2, HLA-A, HPX, HSPA5, HSPA8, IGF1, ITIH1, ITIH2, ITIH3, ITIH4, LAP3, LCP1, LOXL1, LTF, MST1, MYOCD, NRP1, PGLYRP2, PI16, PKM, PM20D1, PPIA, PRDX2, PROS1, PROZ, PSMA7, SAA2-SAA4
Tissue Morphology	4.3307	-1.1220	AMBP, APOA1, BHMT, C5, CAMP, CFH, CRP, HLA-A, HP, HPX, HSPA5, IGF1, MST1, NRP1, PKM, PPIA, PRDX2, PROS1, PSAP, SERPINF1

Table S1. *Cont.*

Ingenuity Canonical Pathways	-log (*p* value)	Activation z-score	Molecules
Molecular Transport	4.3298	-1.7250	ACE, APOA1, APOM, CAMP, CRP, FGG, HLA-A, HP, HPX, HSPA5, HSPA8, IGF1, LBP, LTF, PKM, PPIA, PSAP, SERPINF1
Gastrointestinal Disease, Inflammatory Disease	4.3279		CAMP, CRP, F5, IGF1, LBP, LTF, PKM
Cancer, Organismal Injury and Abnormalities	4.3179	-0.0230	AHSG, AMBP, APOA1, C5, CFH, FGG, IGF1, LTF, MYOCD, NRP1, PKM, PPIA, PRDX2, PSAP, SERPINF1
Inflammatory Disease, Inflammatory Response, Organismal Injury and Abnormalities, Respiratory Disease	4.3161		ACE, AMBP, C5, CAMP, CRP, HLA-A, HSPA5, LBP
Digestive System Development and Function, Gastrointestinal Disease, Hepatic System Development and Function, Hepatic System Disease, Inflammatory Disease, Inflammatory Response, Organ Development, Organismal Injury and Abnormalities	4.3054		APOA1, C5, HLA-A, HP, HPX, HSPA5, IGF1, PPIA
Cellular Development, Embryonic Development, Nervous System Development and Function, Organ Development, Organismal Development, Tissue Development, Visual System Development and Function	4.2967		CFH, IGF1, SERPINF1
Carbohydrate Metabolism, Lipid Metabolism, Small Molecule Biochemistry	4.2967		IGF1, LBP, PSAP
Connective Tissue Disorders, Hematological Disease, Organismal Injury and Abnormalities	4.2907		AMBP, CRP, HPX, IGF1, PPIA

Table S1. *Cont.*

Ingenuity Canonical Pathways	-log (*p* value)	Activation z-score	Molecules
Organismal Development	4.2882		ACE, AMBP, APOA1, BHMT, CFH, COLEC11, HP, HPX, IGF1, LOXL1, MST1, MYOCD, NRP1, PGLYRP2, PPIA, PRDX2, PSAP
Free Radical Scavenging	4.2874	-1.1310	ACE, C5, CRP, IGF1
Cellular Development	4.2874		CFH, IGF1, MYOCD, PKM
Cell-To-Cell Signaling and Interaction, Hematological System Development and Function, Immune Cell Trafficking, Inflammatory Response	4.2668		APOA1, CAMP, LBP, PKM
Cellular Growth and Proliferation, Organismal Development	4.2510		APOA1, CAMP, LTF
Cellular Movement, Embryonic Development	4.2510		APOA1, CAMP, PPIA
Cardiovascular System Development and Function, Cellular Development, Organismal Development, Skeletal and Muscular System Development and Function, Tissue Development	4.2510		CFH, IGF1, MYOCD
Organismal Development, Renal and Urological System Development and Function	4.2510		ACE, C5, CFH, IGF1, MST1, MYOCD, PSAP
Cellular Movement, Hematological System Development and Function, Immune Cell Trafficking, Inflammatory Response	4.2510	-0.9150	APOA1, C2, C5, CFB, CFH, LBP
Cell Death and Survival, Organismal Injury and Abnormalities	4.2418	-1.5840	CRP, HSPA5, IGF1, PPIA, PRDX2, SERPINF1
Dermatological Diseases and Conditions, Organismal Injury and Abnormalities	4.2284		CAMP, CRP, NRP1, PPIA, PSAP

Table S1. *Cont.*

Ingenuity Canonical Pathways	-log (*p* value)	Activation z-score	Molecules
Neurological Disease, Organismal Injury and Abnormalities	4.2255	0.1470	ACE, ACTBL2, APOA1, C5, C9, CEP250, CFB, COL24A1, DYNC1H1, F12, F5, HSPA8, IGF1, ITIH2, ITIH4, LOXL1, NRP1, PGLYRP2, PKM, PM20D1, PPIA, PROS1, PROZ, PSAP, SERPINA10
Lipid Metabolism, Molecular Transport, Small Molecule Biochemistry	4.2204		IGF1, PSAP
Molecular Transport	4.2204		CAMP, HSPA8
Cell Death and Survival	4.2204		CAMP, LTF
Infectious Diseases	4.2204		C5, C9
Infectious Diseases	4.2204		C5, C9
Cell Morphology, Organ Morphology, Organismal Development, Renal and Urological System Development and Function	4.2204		C5, CFH
Cell-To-Cell Signaling and Interaction	4.2204		CAMP, CFH
DNA Replication, Recombination, and Repair	4.2204		CAMP, PPIA
Cell Death and Survival, Organismal Injury and Abnormalities, Renal and Urological Disease	4.2076		IGF1, MST1, PKM
Cell Death and Survival, Nervous System Development and Function	4.2076		C5, IGF1, SERPINF1
Cardiovascular System Development and Function	4.1765		ACE, CFH, IGF1, MYOCD, NRP1, PPIA, PROS1, PSAP
Carbohydrate Metabolism	4.1701	-1.4890	ACE, AHSG, APOA1, C5, HSPA5, IGF1, PKM
Cellular Movement, Renal and Urological System Development and Function	4.1656		APOA1, CAMP, PPIA
Cellular Movement	4.1506	-1.2880	APOA1, C5, CAMP, HP, LBP, MST1, PPIA

Table S1. *Cont.*

Ingenuity Canonical Pathways	-log (*p* value)	Activation z-score	Molecules
Cell-To-Cell Signaling and Interaction, Hematological System Development and Function, Immune Cell Trafficking, Inflammatory Response	4.1481	-2.0000	C5, CAMP, LBP, PPIA
Hereditary Disorder, Ophthalmic Disease, Organismal Injury and Abnormalities	4.1469		C2, C9, CEP250, CFB, CFH, PSAP
Molecular Transport	4.1367	-1.0110	C5, HLA-A, HP, HPX, HSPA5, IGF1, LTF, PPIA, PRDX2
Cancer, Organismal Injury and Abnormalities	4.1232	-0.0230	AHSG, AMBP, APOA1, C5, FGG, IGF1, LTF, MYOCD, NRP1, PKM, PPIA, PRDX2, PSAP, SERPINF1
Organismal Injury and Abnormalities, Renal and Urological Disease	4.1203		ACE, C5, CRP, F5, PPIA
Cell Death and Survival	4.1101	-1.1920	IGF1, NRP1, PKM, PRDX2
Immunological Disease, Inflammatory Disease, Inflammatory Response, Organismal Injury and Abnormalities, Renal and Urological Disease	4.1101		ACE, CFH, CRP, PPIA
Lipid Metabolism, Small Molecule Biochemistry	4.1090	-1.7040	APOA1, APOM, C5, CAMP, HSPA8, IGF1, LBP, LTF, PKM, PSAP
Cellular Movement	4.1085	-1.3420	AHSG, IGF1, LTF, MST1, SERPINF1
Cardiovascular Disease, Organismal Injury and Abnormalities	4.1051		ACE, C5, CFB, CFH, HLA-A, PPIA
Cancer, Organismal Injury and Abnormalities	4.0910		ACE, ACTBL2, ALDOB, APOA1, C2, C5, C9, CAMP, CEP250, CFB, CFH, COL24A1, COLEC11, CRP, DYNC1H1, F5, FGG, HABP2, HLA-A, HP, HPX, HSPA5, HSPA8, IGF1, ITIH1, ITIH2, ITIH3, ITIH4, LAP3, LBP, LCP1, LOXL1, LTF, MST1, MYOCD, NRP1, PI16, PKM, PM20D1, PPIA, PROS1, PSAP, PSMA7, SAA2-SAA4, SERPINF1

Table S1. *Cont.*

Ingenuity Canonical Pathways	-log (*p* value)	Activation z-score	Molecules
Cellular Development, Cellular Growth and Proliferation, Hematological System Development and Function, Lymphoid Tissue Structure and Development	4.0894	-0.4530	CAMP, CRP, HPX, HSPA5, HSPA8, IGF1, LCP1, LTF, PRDX2, Serpina3g (includes others)
Inflammatory Disease, Inflammatory Response, Organismal Injury and Abnormalities, Respiratory Disease	4.0857		ACE, C5, CAMP, CRP, LBP
Hereditary Disorder, Immunological Disease, Organismal Injury and Abnormalities	4.0742		C2, C5, CFB, FGG, IGF1
Cellular Function and Maintenance	4.0660	-1.1540	ACE, APOA1, CAMP, CFH, CRP, PKM, PROS1
Cellular Movement, Hematological System Development and Function, Immune Cell Trafficking, Inflammatory Response	4.0535	-1.2900	APOA1, C5, CAMP, HP, LBP, MST1, PPIA
Cardiovascular System Development and Function, Tissue Development	4.0477		CAMP, LTF, NRP1
Cellular Movement, Hematological System Development and Function, Immune Cell Trafficking, Inflammatory Response	4.0477		APOA1, LCP1, PPIA
Carbohydrate Metabolism, Small Molecule Biochemistry	4.0453		APOA1, CAMP
Hereditary Disorder, Ophthalmic Disease, Organismal Injury and Abnormalities	4.0453		C9, CFH
Cell Death and Survival	4.0453		CAMP, LTF
Hematological Disease	4.0453		C5, HPX

Table S1. C*ont.*

Ingenuity Canonical Pathways	-log (*p* value)	Activation z-score	Molecules
Gastrointestinal Disease, Hepatic System Disease, Organismal Injury and Abnormalities	4.0348		APOA1, C5, CRP, FGG, HP, HPX, PPIA
Cellular Assembly and Organization, Cellular Function and Maintenance	4.0106		C5, CAMP, FGG
Cardiovascular Disease, Organismal Injury and Abnormalities, Renal and Urological Disease	4.0106		C5, CFB, COLEC11
Cell-To-Cell Signaling and Interaction, Hematological System Development and Function, Immune Cell Trafficking, Inflammatory Response	4.0074	-1.9690	C5, CAMP, HLA-A, HPX, IGF1, LBP, LTF, PGLYRP2, PPIA
Cardiovascular Disease	3.9914		APOA1, CRP, F5, FGG, IGF1, MYOCD, PPIA
Cell-To-Cell Signaling and Interaction, Cellular Growth and Proliferation	3.9914	-2.6030	APOA1, C5, CAMP, IGF1, LTF, MST1, PSAP
Cellular Movement	3.9747		CAMP, IGF1, SERPINF1
Cellular Movement, Organismal Injury and Abnormalities, Respiratory Disease	3.9666		C2, C5, CFB, HSPA5
Cardiovascular Disease, Neurological Disease, Organismal Injury and Abnormalities	3.9508		APOA1, F5, IGF1, PROS1
Cellular Compromise	3.9508	1.9250	CFH, HP, HPX, IGF1
Organ Morphology, Organismal Development, Renal and Urological System Development and Function	3.9469		ACE, C5, CFH, COLEC11, HP, IGF1, PSAP

Table S1. C*ont.*

Ingenuity Canonical Pathways	-log (*p* value)	Activation z-score	Molecules
Cellular Development, Cellular Growth and Proliferation, Hematological System Development and Function, Lymphoid Tissue Structure and Development	3.9208	-0.5640	CAMP, CRP, HPX, HSPA5, HSPA8, IGF1, LCP1, LTF, PPIA, PRDX2, Serpina3g (includes others)
Cellular Development, Cellular Growth and Proliferation	3.9208	-0.1560	APOA1, CAMP, CRP, HPX, HSPA5, HSPA8, IGF1, LCP1, LTF, PPIA, PRDX2, Serpina3g (includes others)
Cell Signaling, Inflammatory Response	3.8996		CFB, CFH
Cardiovascular Disease	3.8996		ACE, CRP
Cell Death and Survival	3.8996		CAMP, CFH
Cardiovascular Disease, Organismal Injury and Abnormalities, Skeletal and Muscular Disorders	3.8996		C5, IGF1
Cellular Movement, Hematological System Development and Function, Immune Cell Trafficking, Inflammatory Response	3.8996		C5, MST1
Organismal Injury and Abnormalities	3.8928		C5, CAMP, CFB, CRP, IGF1, LTF
Organismal Injury and Abnormalities, Renal and Urological Disease	3.8761	0.7840	ACE, CFB, CFH, CRP, MST1, PKM, PSAP
Hereditary Disorder, Immunological Disease, Organismal Injury and Abnormalities	3.8761		C2, C5, CFB, FGG, IGF1, PPIA
Cellular Function and Maintenance	3.8729		C5, HP, PROS1

Table S1. *Cont.*

Ingenuity Canonical Pathways	-log (*p* value)	Activation z-score	Molecules
Connective Tissue Disorders, Inflammatory Disease, Inflammatory Response, Organismal Injury and Abnormalities, Skeletal and Muscular Disorders	3.8697	-1.0000	APOA1, C5, CFB, FGG, PKM
Cardiovascular Disease, Organismal Injury and Abnormalities	3.8697		APOA1, CRP, F5, FGG
Respiratory Disease	3.8539		CRP, HSPA8, IGF1, LBP, LCP1, LOXL1, LTF, PRDX2
Hereditary Disorder, Neurological Disease, Organismal Injury and Abnormalities	3.8539		ACE, AHSG, APOA1, C5, C9, CEP250, CRP, DYNC1H1, FGG, HP, HSPA5, HSPA8, IGF1, ITIH3, ITIH4, PKM, PPIA, PRDX2, PSAP, SERPINF1
Metabolic Disease, Organismal Injury and Abnormalities, Renal and Urological Disease	3.8539		ACE, CFH, CRP, PKM
Gastrointestinal Disease, Inflammatory Disease, Inflammatory Response, Organismal Injury and Abnormalities	3.8386		CAMP, CFB, HP, Retnlg, Serpina3g (includes others)
Carbohydrate Metabolism, Molecular Transport, Small Molecule Biochemistry	3.8327	-1.1610	ACE, AHSG, APOA1, HSPA5, IGF1, PKM
Cardiovascular Disease, Cardiovascular System Development and Function, Organismal Injury and Abnormalities	3.8297		ACE, CFH, IGF1, MYOCD, NRP1, PROS1, PSAP
Connective Tissue Disorders, Organismal Injury and Abnormalities, Skeletal and Muscular Disorders, Tissue Morphology	3.8297	-1.9820	C5, CFB, IGF1, PKM, PRDX2

Table S1. *Cont.*

Ingenuity Canonical Pathways	-log (*p* value)	Activation z-score	Molecules
Cell-mediated Immune Response, Cellular Development, Cellular Function and Maintenance, Cellular Growth and Proliferation, Embryonic Development, Hematological System Development and Function, Hematopoiesis, Lymphoid Tissue Structure and Development, Organ Development, Organismal Development, Tissue Development	3.7905	0.8160	CRP, F5, HLA-A, HPX, IGF1, PGLYRP2, PKM, PRDX2, PSAP
Cell Death and Survival	3.7747		CAMP, CRP, IGF1, LTF
Cellular Movement, Hematological System Development and Function, Immune Cell Trafficking, Inflammatory Response	3.7747		C5, CAMP
Lipid Metabolism, Small Molecule Biochemistry	3.7747		LBP, PSAP
Cellular Movement, Hematological System Development and Function, Immune Cell Trafficking, Inflammatory Response	3.7696	-0.2230	APOA1, C5, CAMP, LBP, PPIA
Cell Death and Survival	3.7670	-2.4290	ACE, APOA1, C5, CAMP, CFH, HLA-A, HSPA5, HSPA8, IGF1, LTF, NRP1, PKM, PPIA, PRDX2, PSAP, PSMA7, SERPINF1
Organismal Injury and Abnormalities, Renal and Urological Disease	3.7471		AHSG, AMBP, C5, PKM
Inflammatory Response, Neurological Disease	3.7282	0.5790	C5, CRP, F12, F5, HLA-A, HPX, HSPA5, IGF1
Hereditary Disorder, Neurological Disease, Organismal Injury and Abnormalities	3.7212		ACE, AHSG, C5, C9, CRP, DYNC1H1, FGG, HP, HSPA5, HSPA8, IGF1, ITIH3, ITIH4, PKM, PPIA, PRDX2, PSAP

Table S1. *Cont.*

Ingenuity Canonical Pathways	-log (*p* value)	Activation z-score	Molecules
Lipid Metabolism, Molecular Transport, Small Molecule Biochemistry	3.7190	-1.9020	APOA1, APOM, HSPA8, IGF1, LBP, PSAP
Cell Death and Survival	3.7167	-0.3710	C9, CFB, CFH, HLA-A
Lipid Metabolism, Molecular Transport, Small Molecule Biochemistry	3.7122	-1.0440	APOA1, APOM, BHMT, CRP, HP, IGF1, PKM
Cell-To-Cell Signaling and Interaction	3.7055	-1.1310	C5, CRP, HSPA5, IGF1, PKM, PROS1
Cell-To-Cell Signaling and Interaction, Hematological System Development and Function, Immune Cell Trafficking, Inflammatory Response	3.7033	-1.1030	APOA1, C5, CFH, LCP1
Cellular Development, Cellular Growth and Proliferation, Embryonic Development, Hematological System Development and Function, Hematopoiesis, Lymphoid Tissue Structure and Development, Organ Development, Organismal Development, Tissue Development	3.6990	0.2270	CRP, F5, HLA-A, HPX, IGF1, LCP1, PGLYRP2, PKM, PRDX2, PSAP
Immunological Disease	3.6968	1.0670	C5, CAMP, CFH, HLA-A, HSPA5, LBP, PPIA, PSAP
Endocrine System Disorders, Gastrointestinal Disease, Metabolic Disease, Ophthalmic Disease, Organismal Injury and Abnormalities	3.6904		CRP, IGF1, SERPINF1
Cardiovascular Disease, Organismal Injury and Abnormalities, Skeletal and Muscular Disorders	3.6840		ACE, APOA1, C5, CRP, FGG, PPIA
Molecular Transport	3.6737	0.3990	HLA-A, HP, HPX, LTF

Table S1. *Cont.*

Ingenuity Canonical Pathways	-log (*p* value)	Activation z-score	Molecules
Gastrointestinal Disease, Inflammatory Disease	3.6696		CAMP, CRP, F5, IGF1, LBP, LTF, PKM, Serpina3g (includes others)
Cardiovascular Disease, Cell Death and Survival, Connective Tissue Disorders, Hematological Disease, Hereditary Disorder, Organismal Injury and Abnormalities, Renal and Urological Disease	3.6676		CFB, CFH
Cardiovascular Disease, Hematological Disease, Organismal Injury and Abnormalities, Renal and Urological Disease	3.6676		C2, CFB
Cell Death and Survival, Nervous System Development and Function	3.6676		IGF1, SERPINF1
Lipid Metabolism, Small Molecule Biochemistry	3.6635	-1.6980	APOA1, APOM, C5, HSPA5, IGF1, PRDX2
Cellular Assembly and Organization, Tissue Development	3.6635		ACE, HSPA5, IGF1
Cardiovascular Disease, Connective Tissue Disorders, Hematological Disease, Hereditary Disorder, Organismal Injury and Abnormalities	3.6596		AMBP, CRP, HPX, IGF1
Cellular Function and Maintenance	3.6576	-1.3870	ACE, APOA1, C5, CAMP, PROS1
Organismal Injury and Abnormalities	3.6536	1.0910	ACE, APOA1, C5, CRP, FGG, HP, HPX, HSPA5, IGF1, MYOCD, PPIA
Cancer, Gastrointestinal Disease, Organismal Injury and Abnormalities	3.6364	0.4980	ACE, ACTBL2, AHSG, ALDOB, AMBP, APOA1, BHMT, C2, C5, C8G, C9, CAMP, CEP250, CFB, CFH, COL24A1, COLEC11, CRP, DYNC1H1, F12, F5, FGG, HABP2, HLA-A, HP, HPX, HSPA5, HSPA8, IGF1, ITIH1, ITIH2, ITIH3, ITIH4, LAP3, LBP, LCP1, LOXL1, LTF, MST1, MYOCD, NRP1, PGLYRP2, PI16, PKM, PM20D1, PPIA, PRDX2, PROS1, PROZ, PSAP, PSMA7, SAA2-SAA4, SERPINA10

Table S1. *Cont.*

Ingenuity Canonical Pathways	-log (*p* value)	Activation z-score	Molecules
Connective Tissue Disorders, Organismal Injury and Abnormalities, Tissue Morphology	3.6364		ACE, APOA1, IGF1
Cell Death and Survival, Nervous System Development and Function	3.6364		HLA-A, IGF1, SERPINF1
Post-Translational Modification, Protein Degradation, Protein Synthesis	3.6308	-1.0670	ACE, C5, F12, HABP2, HSPA8, IGF1
Endocrine System Disorders, Metabolic Disease, Organismal Injury and Abnormalities	3.6003		ACE, AHSG, APOA1, CRP, LBP, LTF, PRDX2
Cancer, Organismal Injury and Abnormalities	3.5952		APOA1, BHMT, CFB, CFH, COL24A1, CRP, HLA-A, HP, IGF1, NRP1, PRDX2, SERPINF1
Nutritional Disease	3.5784	1.9820	APOA1, IGF1, LTF, MYOCD, PGLYRP2, PSAP
Cardiovascular Disease	3.5784	-2.0000	C5, CFB, COLEC11, PKM
Organismal Injury and Abnormalities	3.5768	-1.1650	APOA1, BHMT, CFB, CFH, COL24A1, CRP, HLA-A, HP, IGF1, NRP1, PRDX2, PROS1, SERPINF1
Cell Death and Survival, Cell Morphology, Embryonic Development, Nervous System Development and Function, Ophthalmic Disease, Organ Development, Organ Morphology, Organismal Development, Organismal Injury and Abnormalities, Tissue Development, Tissue Morphology, Visual System Development and Function	3.5702		IGF1, SERPINF1
Cardiovascular Disease, Hematological Disease, Metabolic Disease, Organismal Injury and Abnormalities	3.5654		APOA1, CRP, LBP, PPIA

Table S1. *Cont.*

Ingenuity Canonical Pathways	-log (*p* value)	Activation z-score	Molecules
Lipid Metabolism, Molecular Transport, Small Molecule Biochemistry	3.5591	0.7710	AHSG, APOA1, APOM, BHMT, CRP, IGF1, SAA2-SAA4
Hematological System Development and Function, Tissue Morphology	3.5528	0.0720	AMBP, APOA1, C5, CAMP, CFH, CRP, HLA-A, HPX, IGF1, PPIA, PRDX2, PROS1, PSAP
Cancer, Organismal Injury and Abnormalities	3.5361		ACTBL2, APOA1, C2, C5, C8G, C9, CEP250, CFB, CFH, COL24A1, CRP, DYNC1H1, F12, F5, FGG, HLA-A, HP, HPX, HSPA5, IGF1, ITIH3, ITIH4, LBP, LCP1, LOXL1, LTF, MST1, MYOCD, NRP1, PI16, PKM, PM20D1, PPIA, PROS1, PROZ, PSMA7, SERPINF1
Cell-To-Cell Signaling and Interaction, Hematological System Development and Function, Immune Cell Trafficking, Inflammatory Response	3.5317		CRP, LCP1, PRDX2
Molecular Transport, Small Molecule Biochemistry	3.5317		C5, CAMP, F5
Organismal Development	3.5003	-0.5550	APOA1, F12, NRP1, PROS1, SERPINF1
Gastrointestinal Disease, Immunological Disease, Ophthalmic Disease, Organismal Injury and Abnormalities	3.5003		APOA1, CRP, HP, PPIA
Dermatological Diseases and Conditions, Organismal Injury and Abnormalities	3.4935	-1.3530	ACE, AHSG, ALDOB, AMBP, APOA1, BHMT, C2, C5, C9, CAMP, CEP250, CFB, CFH, COL24A1, COLEC11, CRP, DYNC1H1, F12, F5, FGG, HABP2, HLA-A, HP, HPX, HSPA5, HSPA8, IGF1, ITIH1, ITIH2, ITIH3, LBP, LCP1, LTF, MST1, MYOCD, NRP1, PGLYRP2, PI16, PKM, PPIA, PROS1, PSAP, SAA2-SAA4, SERPINA10, SERPINF1

Table S1. *Cont.*

Ingenuity Canonical Pathways	-log (*p* value)	Activation z-score	Molecules
Cancer, Organismal Injury and Abnormalities	3.4935	-0.0420	ACE, ACTBL2, AHSG, ALDOB, AMBP, APOA1, BHMT, C2, C5, C8G, C9, CAMP, CEP250, CFB, CFH, COL24A1, COLEC11, CRP, DYNC1H1, F12, F5, FGG, HABP2, HLA-A, HP, HPX, HSPA5, HSPA8, IGF1, ITIH1, ITIH2, ITIH3, ITIH4, LAP3, LBP, LCP1, LOXL1, LTF, MST1, MYOCD, NRP1, PGLYRP2, PI16, PKM, PM20D1, PPIA, PRDX2, PROS1, PROZ, PSAP, PSMA7, SAA2-SAA4, SERPINA10, SERPINF1
Hereditary Disorder, Organismal Injury and Abnormalities, Skeletal and Muscular Disorders	3.4895		ACE, DYNC1H1, HLA-A, IGF1, LBP, LOXL1, PSAP
Cellular Function and Maintenance	3.4841		C5, CFH
Cell Signaling	3.4841		MST1, NRP1
Carbohydrate Metabolism, Small Molecule Biochemistry	3.4841		CFH, IGF1, ITIH2
Inflammatory Disease, Inflammatory Response, Organismal Injury and Abnormalities, Respiratory Disease	3.4802		APOA1, C5, CRP, HSPA5, IGF1, LTF, PPIA
Cancer, Organismal Injury and Abnormalities	3.4776		ACE, ACTBL2, ALDOB, APOA1, C2, C5, C8G, C9, CAMP, CFB, CFH, COL24A1, COLEC11, CRP, DYNC1H1, F5, FGG, HABP2, HLA-A, HP, HPX, HSPA5, HSPA8, IGF1, ITIH1, ITIH2, ITIH3, ITIH4, LAP3, LCP1, LOXL1, LTF, MST1, MYOCD, NRP1, PKM, PM20D1, PPIA, PROS1, PSAP, PSMA7, SAA2-SAA4, SERPINF1
Developmental Disorder, Hereditary Disorder, Organismal Injury and Abnormalities, Skeletal and Muscular Disorders	3.4763		ACE, HLA-A, LBP, LOXL1, PSAP
Cellular Movement, Hematological System Development and Function, Hypersensitivity Response, Immune Cell Trafficking	3.4750	0.6050	C5, CAMP, LTF, PPIA
Organismal Development	3.4711	-0.9700	ACE, HABP2, IGF1, NRP1, PPIA, SERPINF1
Lipid Metabolism, Small Molecule Biochemistry	3.4685	-1.7680	APOA1, APOM, BHMT, C5, CAMP, HSPA5, HSPA8, IGF1, LTF, PKM, PRDX2

Table S1. *Cont*

Ingenuity Canonical Pathways	-log (*p* value)	Activation z-score	Molecules
Cardiovascular System Development and Function, Tissue Morphology	3.4597		APOA1, CRP, IGF1
Cellular Development, Cellular Growth and Proliferation, Organ Development, Renal and Urological System Development and Function	3.4597		C5, IGF1, MST1
Organismal Injury and Abnormalities, Reproductive System Disease	3.4597		C5, F5, PPIA
Cell Death and Survival, Organismal Injury and Abnormalities	3.4597	-1.5130	CRP, HSPA5, IGF1, PPIA, SERPINF1
Nucleic Acid Metabolism, Small Molecule Biochemistry	3.4437		ACE, APOA1, CFH, HSPA8, PKM
Cardiovascular System Development and Function, Ophthalmic Disease, Organismal Development, Organismal Injury and Abnormalities, Visual System Development and Function	3.4365		IGF1, NRP1, SERPINF1
Inflammatory Disease, Organismal Injury and Abnormalities, Respiratory Disease	3.4260		ACE, C5, F12, HABP2
Cell-To-Cell Signaling and Interaction, Inflammatory Response	3.4191	-1.1810	ACE, APOA1, C5, CAMP, PROS1
Dermatological Diseases and Conditions, Organismal Injury and Abnormalities	3.4145		C5, PPIA, PROS1
Cell Death and Survival	3.4056		CAMP, LTF
Cardiovascular Disease, Cellular Compromise, Organismal Injury and Abnormalities	3.4056		HP, HPX

Table S1. *Cont*

Ingenuity Canonical Pathways	-log (*p* value)	Activation z-score	Molecules
Cellular Growth and Proliferation, Organismal Development	3.4056		CAMP, LTF
Cell Death and Survival	3.4034	2.1800	CFH, HSPA5, HSPA8, IGF1, PRDX2
Cell-To-Cell Signaling and Interaction, Hematological System Development and Function, Immune Cell Trafficking, Inflammatory Response	3.4023	-1.9160	C5, CFH, F5, LTF
Embryonic Development, Organ Development, Organismal Development, Renal and Urological System Development and Function, Tissue Development	3.3936		ACE, C5, CFH, IGF1, MST1, PSAP
Protein Synthesis	3.3915		APOA1, CRP, FGG
Cell Death and Survival, Nervous System Development and Function	3.3915		IGF1, PSAP, SERPINF1
Organismal Injury and Abnormalities, Renal and Urological Disease, Renal and Urological System Development and Function	3.3872		ACE, CFH, COLEC11, HP, MYOCD, PSAP
Cell Death and Survival	3.3716	-0.1820	CAMP, CRP, LTF, PROS1, SERPINF1
Endocrine System Disorders, Gastrointestinal Disease, Metabolic Disease, Organismal Injury and Abnormalities	3.3686		ACE, AMBP, BHMT, C9, COL24A1, CRP, IGF1, SERPINF1
Developmental Disorder, Hereditary Disorder, Organismal Injury and Abnormalities, Skeletal and Muscular Disorders	3.3675		ACE, HLA-A, LOXL1, PSAP
Cardiovascular System Development and Function, Cell-To-Cell Signaling and Interaction	3.3645	0.0000	C5, F12, F5, HSPA5, NRP1

Table S1. Cont

Ingenuity Canonical Pathways	-log (*p* value)	Activation z-score	Molecules
Cancer, Gastrointestinal Disease, Organismal Injury and Abnormalities	3.3645	-1.0670	ACE, ACTBL2, AHSG, ALDOB, AMBP, APOA1, BHMT, C2, C5, C8G, C9, CAMP, CEP250, CFB, CFH, COL24A1, COLEC11, CRP, DYNC1H1, F12, F5, FGG, HABP2, HLA-A, HP, HPX, HSPA5, HSPA8, IGF1, ITIH1, ITIH2, ITIH3, ITIH4, LAP3, LBP, LCP1, LOXL1, LTF, MST1, MYOCD, NRP1, PGLYRP2, PI16, PKM, PM20D1, PPIA, PROS1, PROZ, PSAP, PSMA7, SAA2-SAA4, SERPINA10
Cancer, Organismal Injury and Abnormalities	3.3605		ACE, AHSG, C5, C9, CEP250, COL24A1, CRP, DYNC1H1, F12, F5, FGG, HLA-A, HP, HSPA5, HSPA8, IGF1, LCP1, LOXL1, MST1, MYOCD, NRP1, PGLYRP2, PI16, PKM, PPIA, SERPINF1
Organismal Injury and Abnormalities, Renal and Urological Disease	3.3526		ACE, C5, CRP, F5, MYOCD, PPIA
Cellular Development, Hematological System Development and Function, Lymphoid Tissue Structure and Development	3.3449	-1.0670	APOA1, C5, CAMP, NRP1
Hematological Disease, Infectious Diseases, Neurological Disease, Organismal Injury and Abnormalities	3.3344		C9, CFB
Cell Death and Survival	3.3344		CRP, LTF
Cell-To-Cell Signaling and Interaction	3.3344		NRP1, PKM
Cell Death and Survival	3.3107		C5, IGF1, PSAP, SERPINF1
Lipid Metabolism, Molecular Transport, Small Molecule Biochemistry	3.3019	-1.0720	APOA1, APOM, BHMT, CRP, HP, IGF1, PKM, PSAP
Psychological Disorders	3.3010		ACE, AMBP, CRP, IGF1, ITIH4, PSMA7
Protein Synthesis	3.2907	0.2770	AHSG, APOA1, APOM, BHMT, CRP, FGG, IGF1, PPIA
Organismal Injury and Abnormalities, Respiratory Disease	3.2882		CRP, IGF1, LBP, LTF, PRDX2

Table S1. Co*nt*

Ingenuity Canonical Pathways	-log (*p* value)	Activation z-score	Molecules
Nervous System Development and Function, Tissue Development	3.2676		C5, IGF1
Hematological Disease	3.2676		FGG, PPIA
Cellular Movement, Hematological System Development and Function, Immune Cell Trafficking, Inflammatory Response	3.2676		APOA1, C5
Organismal Injury and Abnormalities, Respiratory Disease	3.2668		HSPA8, IGF1, LCP1, LTF, PPIA
Cancer, Organismal Injury and Abnormalities	3.2636		ACTBL2, APOA1, C2, C5, C8G, C9, CEP250, CFB, CFH, COL24A1, COLEC11, CRP, DYNC1H1, F5, HLA-A, HP, HPX, HSPA5, IGF1, ITIH3, LBP, LCP1, LOXL1, LTF, MST1, MYOCD, NRP1, PI16, PKM, PM20D1, PPIA, PSMA7, SERPINF1
Cancer, Organismal Injury and Abnormalities	3.2588		ACE, AHSG, ALDOB, AMBP, APOA1, BHMT, C2, C5, C8G, CEP250, CFB, CFH, COL24A1, COLEC11, CRP, DYNC1H1, F12, F5, FGG, HABP2, HLA-A, HPX, IGF1, ITIH1, ITIH2, ITIH3, ITIH4, LAP3, LBP, LCP1, LOXL1, LTF, MST1, MYOCD, NRP1, PGLYRP2, PI16, PKM, PM20D1, PPIA, PRDX2, PROS1, PROZ, PSAP, PSMA7, SAA2-SAA4, SERPINF1
Immunological Disease	3.2557		ALDOB, C5, LTF, PPIA
Protein Degradation, Protein Synthesis	3.2526	0.0240	ACE, AMBP, APOA1, C5, F12, HABP2, HSPA5, HSPA8, IGF1
Infectious Diseases	3.2464		C5, CRP, F5
Cell Death and Survival	3.2358	-0.4090	APOA1, C5, C9, HLA-A, HSPA5, IGF1, NRP1, PM20D1, PRDX2, SERPINF1
Cancer, Gastrointestinal Disease, Hepatic System Disease, Organismal Injury and Abnormalities	3.2299		ALDOB, APOA1, BHMT, C9, F5, FGG, HP, HSPA5, HSPA8, IGF1, LAP3, MYOCD, PKM, PROZ

Table S1. *Cont*

Ingenuity Canonical Pathways	-log (*p* value)	Activation z-score	Molecules
Cancer, Organismal Injury and Abnormalities	3.2125		ACE, ACTBL2, ALDOB, APOA1, C2, C5, C8G, C9, CAMP, CFB, CFH, COL24A1, COLEC11, CRP, DYNC1H1, F5, FGG, HABP2, HLA-A, HP, HPX, HSPA5, HSPA8, IGF1, ITIH1, ITIH2, ITIH3, ITIH4, LAP3, LCP1, LOXL1, LTF, MST1, MYOCD, NRP1, PKM, PM20D1, PPIA, PROS1, PSAP, PSMA7, SAA2-SAA4
Cell Death and Survival	3.2104	1.8660	ACE, APOA1, C5, CAMP, CFB, CFH, CRP, DYNC1H1, HSPA5, HSPA8, IGF1, ITIH4, LTF, MST1, MYOCD, NRP1, PKM, PPIA, PRDX2, PROS1, PSAP, SERPINF1
Cancer, Organismal Injury and Abnormalities	3.2090	-1.0000	ACE, AHSG, C5, C9, CEP250, COL24A1, CRP, DYNC1H1, F12, F5, FGG, HLA-A, HP, HSPA5, HSPA8, IGF1, LCP1, LOXL1, MST1, MYOCD, NRP1, PGLYRP2, PI16, PKM, PPIA, SERPINF1
Cell Death and Survival	3.2090	0.0390	CAMP, CRP, IGF1, LTF, SERPINF1
Cell Death and Survival	3.2076		CRP, IGF1, LTF
Cancer, Gastrointestinal Disease, Inflammatory Disease, Organismal Injury and Abnormalities	3.2076		ALDOB, BHMT, C9
Neurological Disease, Organismal Injury and Abnormalities	3.2076		HP, HPX, SERPINF1
Amino Acid Metabolism, Molecular Transport, Small Molecule Biochemistry	3.2062		C5, CFH
Connective Tissue Development and Function, Skeletal and Muscular System Development and Function	3.2062		IGF1, SERPINF1
Cell Death and Survival, Connective Tissue Development and Function, Skeletal and Muscular System Development and Function	3.2062		IGF1, LTF

Table S1. C*ont*

Ingenuity Canonical Pathways	-log (*p* value)	Activation z-score	Molecules
Cancer, Organismal Injury and Abnormalities, Reproductive System Disease	3.1959		ALDOB, APOA1, C2, C5, C9, CFB, CFH, COL24A1, CRP, DYNC1H1, F5, HLA-A, HSPA5, HSPA8, IGF1, ITIH2, LCP1, LTF, MST1, MYOCD, NRP1, PKM, PM20D1, PROS1, PSAP, PSMA7
Cellular Movement, Hematological System Development and Function, Hypersensitivity Response, Immune Cell Trafficking, Inflammatory Response	3.1891		C5, CAMP, PPIA
Cardiovascular Disease, Organismal Injury and Abnormalities	3.1891		APOM, C5, CRP
Developmental Disorder, Hereditary Disorder, Metabolic Disease, Organismal Injury and Abnormalities, Respiratory Disease	3.1891		CRP, IGF1, LTF
Cellular Movement, Hematological System Development and Function, Immune Cell Trafficking, Inflammatory Response	3.1811	-2.3690	APOA1, C5, CAMP, CFH, MST1, SERPINF1
Cell-To-Cell Signaling and Interaction, Inflammatory Response	3.1726		APOA1, C5, CAMP, CRP
Cellular Movement	3.1726	-0.1300	IGF1, NRP1, PRDX2, SERPINF1
Cardiovascular Disease, Hematological Disease, Hereditary Disorder, Metabolic Disease, Organismal Injury and Abnormalities	3.1701		APOA1, CRP, PPIA
Cancer, Organismal Injury and Abnormalities, Reproductive System Disease	3.1681		APOA1, C2, C9, CAMP, CEP250, COL24A1, DYNC1H1, F5, HLA-A, HP, HSPA5, IGF1, ITIH3, LBP, LTF, MST1, MYOCD, PI16, PKM, PM20D1, PPIA, PSMA7, SERPINF1

Table S1. C*ont*

Ingenuity Canonical Pathways	-log (*p* value)	Activation z-score	Molecules
Inflammatory Disease, Inflammatory Response, Neurological Disease, Organismal Injury and Abnormalities	3.1637	0.5790	C5, CRP, F12, HLA-A, HPX, HSPA5, IGF1
Cell-To-Cell Signaling and Interaction, Cellular Movement, Hematological System Development and Function, Immune Cell Trafficking, Inflammatory Response	3.1518		APOA1, C5, CAMP
Dermatological Diseases and Conditions, Organismal Injury and Abnormalities	3.1494		C5, PPIA
Dermatological Diseases and Conditions, Organismal Injury and Abnormalities	3.1494		C5, PPIA
Dermatological Diseases and Conditions, Gastrointestinal Disease, Immunological Disease, Organismal Injury and Abnormalities	3.1494		C5, PPIA
Endocrine System Disorders, Organismal Injury and Abnormalities	3.1494		APOA1, PRDX2
Lipid Metabolism, Small Molecule Biochemistry	3.1494		APOA1, APOM
Cancer, Gastrointestinal Disease, Hepatic System Disease, Organismal Injury and Abnormalities	3.1421		BHMT, ITIH4, MST1, PKM
Carbohydrate Metabolism, Lipid Metabolism, Small Molecule Biochemistry	3.1397	-2.2190	APOA1, C5, HSPA5, IGF1, PRDX2

Table S1. *Cont*

Ingenuity Canonical Pathways	-log (*p* value)	Activation z-score	Molecules
Embryonic Development, Organ Development, Organismal Development, Respiratory System Development and Function, Tissue Development	3.1343		ACE, IGF1, MYOCD
Cellular Growth and Proliferation, Tissue Development	3.1290	-1.5160	C5, CAMP, HSPA5, IGF1, ITIH4, LTF, MST1, SERPINF1
Cell Death and Survival, Organismal Injury and Abnormalities	3.1290	2.0420	ACE, CAMP, HSPA5, IGF1, ITIH4, MST1, PKM, SERPINF1
Post-Translational Modification	3.1290	-2.0720	APOA1, CAMP, CRP, HSPA8, IGF1, MST1, NRP1, PPIA, PSAP
Molecular Transport, Protein Trafficking	3.1226		FGG, IGF1, PPIA, PSAP
Hematological System Development and Function, Organismal Functions	3.1163		ACE, C5, MYOCD
Cellular Development, Hematological System Development and Function, Lymphoid Tissue Structure and Development	3.1124	-1.4320	APOA1, C5, CAMP, LCP1, NRP1
Cancer, Gastrointestinal Disease, Organismal Injury and Abnormalities	3.1002		AHSG, C2, C8G, C9, CAMP, CEP250, CFB, CFH, COL24A1, CRP, DYNC1H1, F12, F5, HLA-A, HSPA8, IGF1, ITIH2, ITIH3, ITIH4, LCP1, LTF, MYOCD, PGLYRP2, PI16, PKM, PM20D1, PPIA, PROS1, PROZ, PSMA7, SAA2-SAA4
Organismal Injury and Abnormalities	3.0953		C5, CFB
Dermatological Diseases and Conditions, Hematological Disease, Immunological Disease, Organismal Injury and Abnormalities	3.0953		C5, PPIA
Cellular Function and Maintenance	3.0926		AMBP, APOA1, COLEC11, HP, HPX
Cell Death and Survival	3.0830	1.5440	CAMP, CFH, HSPA5, IGF1, PKM, SERPINF1

Table S1. *Cont*

Ingenuity Canonical Pathways	-log (*p* value)	Activation z-score	Molecules
Cell-To-Cell Signaling and Interaction, Hematological System Development and Function, Immune Cell Trafficking, Inflammatory Response	3.0820		C5, CFH, NRP1
Hematological Disease, Hereditary Disorder, Organismal Injury and Abnormalities	3.0820		F12, F5, FGG
Cancer, Gastrointestinal Disease, Organismal Injury and Abnormalities	3.0696		ACE, ACTBL2, AHSG, ALDOB, AMBP, APOA1, BHMT, C2, C5, C8G, C9, CAMP, CEP250, CFB, CFH, COL24A1, COLEC11, CRP, DYNC1H1, F12, F5, FGG, HABP2, HLA-A, HPX, HSPA5, HSPA8, IGF1, ITIH1, ITIH2, ITIH3, ITIH4, LAP3, LBP, LCP1, LOXL1, LTF, MST1, MYOCD, NRP1, PGLYRP2, PI16, PKM, PM20D1, PPIA, PROS1, PROZ, PSMA7, SAA2-SAA4
Cancer, Endocrine System Disorders, Organismal Injury and Abnormalities	3.0600		ACE, AHSG, ALDOB, AMBP, APOA1, BHMT, C2, C5, C8G, CEP250, CFB, CFH, COL24A1, COLEC11, CRP, DYNC1H1, F12, F5, FGG, HABP2, HLA-A, HP, HPX, HSPA5, IGF1, ITIH1, ITIH2, ITIH3, ITIH4, LAP3, LBP, LCP1, LOXL1, LTF, MST1, NRP1, PGLYRP2, PI16, PKM, PM20D1, PPIA, PRDX2, PROS1, PROZ, PSAP, PSMA7, SAA2-SAA4, SERPINF1
Cancer, Organismal Injury and Abnormalities, Respiratory Disease	3.0600	-0.7140	ACE, AHSG, ALDOB, APOA1, C9, CAMP, CEP250, CFH, COL24A1, CRP, F5, FGG, HLA-A, HSPA5, HSPA8, IGF1, ITIH1, ITIH2, ITIH3, LAP3, LCP1, LTF, MYOCD, NRP1, PKM, PPIA, PRDX2, PROS1, PSAP, SAA2-SAA4
Cell-To-Cell Signaling and Interaction, Cellular Growth and Proliferation	3.0482		C5, CAMP, IGF1
Post-Translational Modification, Protein Degradation, Protein Synthesis	3.0467		ACE, C5, F12, HABP2, HSPA8
Cardiovascular System Development and Function, Organismal Development	3.0467	-0.7040	HABP2, IGF1, NRP1, PPIA, SERPINF1

Table S1. *Cont*

Ingenuity Canonical Pathways	-log (*p* value)	Activation z-score	Molecules
Cell Morphology, Embryonic Development, Nervous System Development and Function, Organ Development, Organ Morphology, Organismal Development, Tissue Development, Visual System Development and Function	3.0458		CFH, IGF1, LOXL1, SERPINF1
Cellular Movement	3.0448		IGF1, PPIA
Hereditary Disorder, Organismal Injury and Abnormalities	3.0448		ACE, F12
Immunological Disease, Inflammatory Disease, Inflammatory Response, Neurological Disease, Organismal Injury and Abnormalities	3.0448		CRP, IGF1
Carbohydrate Metabolism, Lipid Metabolism, Small Molecule Biochemistry	3.0448		IGF1, LBP
Cardiovascular Disease, Organismal Injury and Abnormalities	3.0448		ACE, MYOCD
Cardiovascular System Development and Function, Cellular Development, Cellular Growth and Proliferation, Embryonic Development, Organ Development, Organismal Development, Skeletal and Muscular System Development and Function, Tissue Development	3.0448		HSPA5, IGF1
Cell-To-Cell Signaling and Interaction, Cellular Movement, Immune Cell Trafficking	3.0448		C5, CAMP
Auditory Disease, Neurological Disease	3.0315		CEP250, IGF1, PSAP

Table S1. *Cont*

Ingenuity Canonical Pathways	-log (*p* value)	Activation z-score	Molecules
Psychological Disorders	3.0278		ACE, AMBP, CRP, IGF1, ITIH1, ITIH4, PSMA7
Cell-To-Cell Signaling and Interaction, Cellular Movement, Hematological System Development and Function, Immune Cell Trafficking	3.0273	-1.1540	APOA1, C5, CAMP, PGLYRP2
Lipid Metabolism, Molecular Transport, Small Molecule Biochemistry	3.0237	-0.7880	APOA1, APOM, BHMT, CRP, HP, PKM
Cancer, Gastrointestinal Disease, Organismal Injury and Abnormalities	3.0195		ALDOB, BHMT, C9, CEP250, COLEC11, F5, LBP
Cancer, Organismal Injury and Abnormalities	3.0177		ACE, ACTBL2, AHSG, ALDOB, AMBP, BHMT, C2, C5, C8G, CEP250, CFB, CFH, COL24A1, COLEC11, CRP, DYNC1H1, F12, F5, FGG, HABP2, HLA-A, HPX, HSPA8, IGF1, ITIH1, ITIH2, ITIH3, ITIH4, LAP3, LBP, LCP1, LOXL1, LTF, MST1, MYOCD, NRP1, PGLYRP2, PI16, PKM, PM20D1, PPIA, PRDX2, PROS1, PROZ, PSAP, PSMA7, SAA2-SAA4, SERPINA10, SERPINF1
Organismal Injury and Abnormalities, Renal and Urological Disease	3.0155		ACE, IGF1, PSAP
Connective Tissue Disorders, Organismal Injury and Abnormalities	3.0092		ACE, APOA1, HSPA5, IGF1
Cardiovascular Disease, Hematological Disease, Organismal Injury and Abnormalities	15.4763	0.5450	C2, C5, CFB, CFH, CRP, F12, F5, FGG, HABP2, MYOCD, PPIA, PRDX2, PROS1, PROZ, SERPINA10
Inflammatory Disease	13.3585		ACE, AHSG, APOA1, APOM, BHMT, C2, C5, CAMP, CFB, CFH, CRP, F5, HLA-A, HP, HSPA5, HSPA8, IGF1, LAP3, LBP, LCP1, LTF, NRP1, PKM, PPIA, PRDX2, Serpina3g (includes others)
